# Biosignal-Based Human–Machine Interfaces for Assistance and Rehabilitation: A Survey

**DOI:** 10.3390/s21206863

**Published:** 2021-10-15

**Authors:** Daniele Esposito, Jessica Centracchio, Emilio Andreozzi, Gaetano D. Gargiulo, Ganesh R. Naik, Paolo Bifulco

**Affiliations:** 1 Department of Electrical Engineering and Information Technologies, Polytechnic and Basic Sciences School, University of Naples “Federico II”, 80125 Naples, Italy; daniele.esposito@unina.it (D.E.); jessica.centracchio@unina.it (J.C.); emilio.andreozzi@unina.it (E.A.); paolo.bifulco@unina.it (P.B.); 2 School of Engineering, Design and Built Environment, Western Sydney University, Penrith, NSW 2747, Australia; g.gargiulo@westernsydney.edu.au; 3 The MARCS Institute, Western Sydney University, Penrith, NSW 2751, Australia; 4 The Adelaide Institute for Sleep Health, Flinders University, Bedford Park, SA 5042, Australia

**Keywords:** Human–Machine Interface, biosignals, assistive technology, rehabilitation, prosthetic control, robotic control, virtual reality control, gesture recognition, communication, smart environment control

## Abstract

As a definition, Human–Machine Interface (HMI) enables a person to interact with a device. Starting from elementary equipment, the recent development of novel techniques and unobtrusive devices for biosignals monitoring paved the way for a new class of HMIs, which take such biosignals as inputs to control various applications. The current survey aims to review the large literature of the last two decades regarding biosignal-based HMIs for assistance and rehabilitation to outline state-of-the-art and identify emerging technologies and potential future research trends. PubMed and other databases were surveyed by using specific keywords. The found studies were further screened in three levels (title, abstract, full-text), and eventually, 144 journal papers and 37 conference papers were included. Four macrocategories were considered to classify the different biosignals used for HMI control: biopotential, muscle mechanical motion, body motion, and their combinations (hybrid systems). The HMIs were also classified according to their target application by considering six categories: prosthetic control, robotic control, virtual reality control, gesture recognition, communication, and smart environment control. An ever-growing number of publications has been observed over the last years. Most of the studies (about 67%) pertain to the assistive field, while 20% relate to rehabilitation and 13% to assistance and rehabilitation. A moderate increase can be observed in studies focusing on robotic control, prosthetic control, and gesture recognition in the last decade. In contrast, studies on the other targets experienced only a small increase. Biopotentials are no longer the leading control signals, and the use of muscle mechanical motion signals has experienced a considerable rise, especially in prosthetic control. Hybrid technologies are promising, as they could lead to higher performances. However, they also increase HMIs’ complexity, so their usefulness should be carefully evaluated for the specific application.

## 1. Introduction

A Human–Machine Interface (HMI) establishes a connection between a person and a device: sensors decipher human intentions and control machine actions, usually allowing real-time, bidirectional interactions. In a broad sense, we can consider the PC keyboard and monitor or even a simple switch as HMIs. Modern HMIs are software-based and replace manually activated controls, providing sophisticated interaction with machinery. HMIs are widely used in industrial control systems, automotive, aviation, military, etc. [1]. User movements, or movement intention, are typically used to interpret the person’s will to operate a device. To this end, multiple and varied sensors have been developed to monitor specific user’s activities, such as hand, eyes, joint, limbs movements, etc. Rotational, displacement, force, pressure, strain, acceleration, and inertial sensors can detect a user’s motion when opportunely connected to the body [1,2,3]. Since muscles generate human motion, physiological signal related to muscle contraction such as electromyography (EMG), mechanomyography (MMG), and force myography (FMG) can be successfully used for HMI [3,4]. Recently, electroencephalography (EEG), able to detect motor brain function, has increasingly been proposed as HMI control signals (these techniques are referred to as Brain–Computer Interfaces (BCIs) [5,6,7,8]). Human movement can also be captured by cameras (the so-called image-based HMIs), which do not require any physical contact with the user [9,10]. Different sensors can be combined to obtain greater sensitivity and specificity in recognizing the user’s intention. Therefore, we can define such mix as hybrid HMI control [11,12,13].

Signals from sensors typically need to be processed for robust recognition of the user’s intention. This processing can widely vary in complexity and ranges from simple thresholding to complex machine learning techniques. Today, machine learning has developed into a scientific branch of its own. For this reason, this paper does not explicitly review this topic.

Most HMIs provide feedback to the user offering visual, acoustic, tactile sensations, which help interaction [14,15,16]. Feedback provides information on the controlled system’s performance to the user. It can consist of a simple light or sound indication or graphical representations or create complex visual–acoustic experiences like virtual reality or tactile sensations (e.g., via vibrations or forces delivery) such as in haptic devices.

In the medical field, biosignal-based HMIs have increasingly been used for assistance and rehabilitation. An “assistive technology” is any system or object used to enhance, maintain, or improve the capabilities of a disabled individual and, more generally, any technology that allows accomplishing something that generally cannot be accomplished. “Rehabilitation” is defined as the physical restoration of a sick or disabled person by therapeutic measures and re-education to participate in everyday life activities within the limitations of the person’s physical disability [17]. People with severe disabilities enormously benefit from using these new HMIs for assistance and rehabilitation purposes. Clear examples are subjects with paraplegia or quadriplegia; those affected by neuromuscular disorders [5,18,19,20] such as Muscular Dystrophy (MD), Amyotrophic Lateral Sclerosis (ALS), or Multiple Sclerosis (MS); people with Spinal Cord Injury (SCI) or Cerebral Palsy (CP); or even stroke survivors and amputees. Literature reviews in these fields are currently limited to particular applications (e.g., prosthetic control, BCI, exergaming, etc.) or focused on specific biosignals. Table 1 provides a summary of some recent surveys regarding assistive and rehabilitative HMIs. As an example, Mohebbi et al. [21] proposed a review about human–robot interaction in assistive and rehabilitation robotics, while Frisoli et al. [22] focused on wearable technologies and, in particular, on a robotic exoskeleton for assistance in performing activities of daily living (ADL). Baniqued et al. [7] presented a review study on BCI robotics for motor rehabilitation of hand movements after stroke. Different surveys such as [9,10,23,24,25,26,27,28] focused specifically on the state-of-the-art and control strategies of upper limb prostheses, while further reviews presented exergaming applications for rehabilitation neuromotor functions [29,30,31,32].

To the best of our knowledge, a broad overview of the current research trends on assistive and rehabilitation HMIs is missing. The current survey aims to review the extensive literature of the last two decades regarding biosignal-based HMIs for assistance and rehabilitation to outline state-of-the-art and identify emerging technologies and potential future research trends.

## 2. Survey Method

Over four months (April 2021 to July 2021), we surveyed Google Scholar, Scopus, PubMed, IEEE Xplore, MDPI, Frontiers, and ScienceDirect to identify HMIs with applications as assistive technology and/or rehabilitation.

Using the following keywords: “Assistive HMI”, “Rehabilitation HMI”, “Prosthetic control”, “Exoskeleton control”, “Assistive robot”, “Rehabilitation robot”, and “Exergaming AND rehabilitation”, we restricted our search to the last two decades, including only papers written in English and fully peer-reviewed.

The initially selected papers were then individually screened in three levels: (1) title, (2) abstract, and (3) full-text, to verify the research presented, the results obtained, and their applications (even those potential). Any paper that did not appear to use biosignals to control HMIs for assistive or rehabilitation was discarded. This process produced 144 journal papers and 37 conference papers.

Four macrocategories were considered to classify the biosignals used as a control for HMIs: (1) *biopotential* (EMG, EEG, etc.), (2) *muscle mechanical motion* (gross muscle motion, muscle vibrations, muscle–tendons movements), (3) *body motion* (limb or joint motion, hand gesture, eye movements), and (4) *hybrid* (a combination of two or more different biosignals).

The type of sensor(s), the site(s) of its/their application, and the HMI target(s) (*prosthetic control, robotic control, virtual reality control, gesture recognition, communication, smart environment control*) was reported for each study.

To facilitate reading, it is worth highlighting briefly the definitions of the primary devices for assistive and rehabilitative purposes that have emerged from the survey:

- *Prosthesis*: an assistive device designed to replace a missing part of the body or to make a part of the body work better. Prosthetic devices commonly replace the diseased or missing eyes, arms, hands, legs, or joints [9,10,23,24,25,26,27,28].
- *Exoskeleton*: an assistive/rehabilitation device that connects with the human body in a wearable way and can control the movement of joints (leg, wrist, hand, fingers), providing the person with the support and energy necessary to perform a movement (hand closing/opening, walking, etc.) [34,35,36].
- *Robotic arm*: a type of mechatronic arm with functions similar to a human arm, which can provide assistance to the person in performing tasks (grabbing and moving objects, etc.) and can also be used as a rehabilitation device [37,38].
- *Smart wheelchair*: a power wheelchair to which computers, sensors, and assistive technology are attached [39,40,41].
- *Smart environment*: environment equipped with intelligent technologies capable of monitoring and assisting the health of people who have difficulty living independently at home [42,43].
- *Exergaming*: a specific type of serious game (not designed for pure entertainment) is the so-called exergame: a human-activated video game that tracks the user’s gestures or movements and simulates them into a connected screen. It can be used as a potential rehabilitation tool to increase physical activity and improve health and physical function in patients with neuromuscular diseases [29,30,31,32].

**Figure 1:**
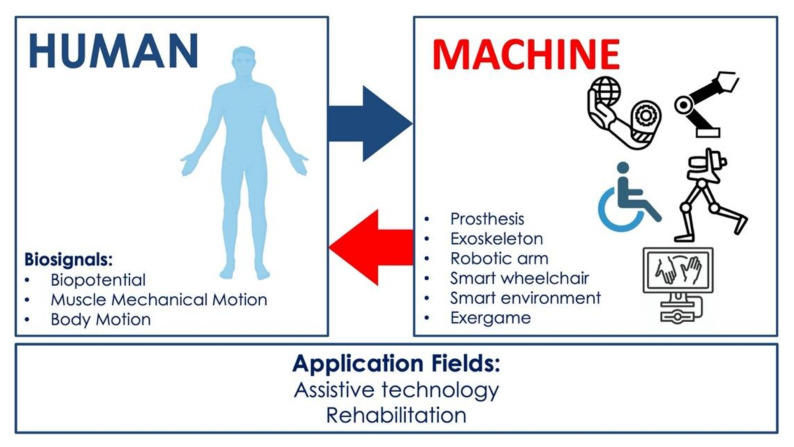
Focus of the survey: biosignal-based HMIs with assistive and rehabilitation purposes.

Figure 1 shows a graphical representation of the focus of this survey, highlighting the kind of biosignal-based control and the primary devices that realize HMIs for assistance and rehabilitation.

## 3. HMI Control Strategies

### 3.1. HMI Control Based on Biopotentials

A large group of HMIs is based on the acquisition of biopotentials, such as electroencephalogram (EEG), electromyogram (EMG), electroneurogram (ENG), electrooculogram (EOG), and electroretinogram (ERG), which are generated by electrical sources in the human body [44]. Hence, they reflect the function of some organs (e.g., brain, muscles, eyes) in the form of electrical activity, providing relevant information about them [44,45]. For this reason, biopotentials are used as control signals in many biomedical HMI applications [25,42,46,47,48,49,50,51,52,53,54,55,56,57,58,59]. These biosignals have their origin in electrophysiological phenomena associated with biochemical events occurring at a cellular level. In detail, some tissues (e.g., nervous, muscular) are composed of excitable cells. At rest, an excitable cell exhibits a transmembrane potential difference in response to a diffusion gradient. Indeed, electrically, the cell membrane works like a leaky capacitor since it consists of a thin insulating layer that separates charge distributions in two conductive media, the intracellular and extracellular environments.

Furthermore, within the dielectric cellular material, the presence of ion-specific passive channels, i.e., pores, results in different ionic concentrations, which drive leakage currents to flow across the membrane. The charge and discharge current of the membrane capacitance is opposed to these ionic currents. At equilibrium, the net current flow is zero. Therefore, the resting potential is achieved. It is maintained by the action of the well-known sodium–potassium pump that actively transports sodium and potassium ions against the electrochemical gradient under the consumption of energy in the form of ATP. When properly stimulated, the excitable cell produces an action potential representing an all-or-none event. It starts only if the transmembrane potential exceeds a threshold value in a specific time interval and travels without attenuation at constant conduction velocity. Due to the electrical activity of many excitable cells, the equivalent current propagates through the surrounding volume conductor, represented by biological tissues, up to the body surface. Consequently, if electrodes are placed in the correct positions on the skin or within underlying tissues, a biopotential representing the spiking cell/tissue phenomenon can be measured [44,45,60,61].

#### 3.1.1. EEG-Based HMIs

The EEG signal originates from the electrical activity of neurons in the brain cortex. To obtain a perceptible signal on the scalp, large populations of neurons must depolarize and repolarize simultaneously in the brain cortex. This synchronization generates the classic oscillations or waves (delta, theta, alpha, beta, and gamma) found in the EEG. In general, some oscillations (as the μ waves of the motor cortex) correspond to an idle rhythm, that is, a state of inactivity. For example, when a person performs or even thinks of acting (such as clench a fist), the idle rhythm is disrupted (e.g., event-related desynchronization) and replaced by a smaller high-frequency signal. Some EEG electrodes can detect this, and this information can be used to give commands to a device. Event-related potentials (e.g., Steady-State Visually Evoked Potentials (SSVEPs), P300) and slow cortical potentials can also be used to implement HMIs. EEG can be classified into two main types: invasive and noninvasive. Noninvasive EEG is commonly recorded by placing electrodes on the scalp.

On the other hand, invasive EEG is acquired intracranially, and it is generally referred to as intracranial EEG (iEEG). It includes both electrocorticogram (ECoG), which is performed by placing electrodes directly onto the brain surface to record electrical activity from the cerebral cortex, and the EEG signal acquired by means of depth electrodes to record electrical activity from deep brain regions, as the stereo-electroencephalogram (sEEG). Scalp EEG amplitude is much lower than other noninvasive biopotentials, and there are issues for electrode fixation and endurance [44,60,61].

Many HMIs are based on the acquisition of the EEG signal, mostly of noninvasive type. Specifically, these technologies are referred to as Brain–Machine Interfaces (BMIs) or Brain–Computer Interfaces (BCIs), since they provide an alternative interaction pathway with the surrounding environment by capturing brain waves and translating them into control signals or commands for an external device [62]. Generally, after a preprocessing phase, some meaningful features are extracted from the acquired EEG signals, and then a classification step is performed to interpret human intention. Afterward, each class is associated with a specific command. In this context, the user is often required to focus on a cognitive task (e.g., motor imagery), or an external stimulation (e.g., visual, auditory, somatosensory) is provided to induce a brain response. The resultant EEG signals are Event-Related Potentials (ERPs), the most commonly used brain waves in a BMI/BCI system [63]. For this reason, in recent years, EEG-based HMIs have found strong application in the assistance and rehabilitation field. Indeed, they represent a powerful tool for people with severe motor disabilities, who need to assist or restore their muscle function or even replace a missing limb, thus improving their quality of life. Clear examples are subjects affected by neuromuscular disorders, such as MD, ALS, MS, SCI, and CP, or even poststroke patients and amputees [5,18,19,20]. In this scenario, two main targets can be identified: robotic control [14,15,34,49,50,64,65,66,67,68,69] and prosthetic control [46,47,48,69,70,71,72,73,74,75,76,77,78].

Regarding robotic control, Song et al. proposed an efficient EEG-based method to control an upper-limb assist robot to help paralysed people perform practical activities or rehabilitation exercises. After applying a visual stimulus, the related EEG signal is classified to decode the human motor intention [49]. Similarly, Korovesis et al. developed a BCI-based system that allows the user to control the motion of a robot vehicle by converting eyes opening and closing into binary sequences, then associating then with different motor commands [50]. Antoniou et al. presented a BCI adopting an eye movement recognition method for hand-free wheelchair control [64]. Xu et al. proposed a motor imagery-based BCI system for teleoperation with tactile feedback to remotely control a robot arm in grasping and reaching tasks [14]. In this context, different studies that use EEG signals for exoskeleton control should be mentioned. Liang et al. extracted some features from EEG signals related to the shoulder’s joint flexion and extension movement. They demonstrated the existence of a relationship between changes in EMG and EEG signals, thus showing feasible to estimate from EEG the minimum torque for controlling an upper-limb exoskeleton robot [34]. He et al. demonstrated the feasibility of decoding joint kinematics and sEMG patterns from scalp EEG to control a powered exoskeleton for gait rehabilitation after stroke [35]. Moreover, Tang et al. proved the effectiveness of a BMI based on Event-Related Desynchronization/Synchronization (ERD/ERS) for controlling an upper-limb exoskeleton, which can assist people in daily living activities [79]. Randazzo et al. designed “mano”, a wearable hand exoskeleton for both assistance and neurorehabilitation purposes. In particular, it exploits a motor imagery-based BMI, which can decode flexion and extension of each finger [80]. Again, Li et al. proposed a BMI-controlled upper limb exoskeleton. The system comprises three main components: EEG signal acquisition and classification into motion commands in task space, redundant motion planning to transform motion commands in joint space, and adaptative neural control to perform manipulation tasks. In detail, visual stimuli are presented to the subject to induce brain signals (i.e., SSVEPs). Then, they are translated into motion commands for controlling the cursor on a computer screen. Afterward, cursor control in task space is converted into exoskeleton control in joint space [81].

Furthermore, López-Larraz et al. presented a BCI system to control an ambulatory exoskeleton for gait rehabilitation of SCI patients, which can benefit from an assist-as-needed paradigm based on the measurement of the stiffness parameter [67]. Xu et al. [36] proposed a BCI system to control an ankle–foot exoskeleton for stroke rehabilitation, which was proven effective for inducing cortical neuroplasticity. Kwak et al. [82] developed an EEG-controlled lower limb exoskeleton for rehabilitation purposes that is based on SSVEPs. Finally, Araujo et al. presented HERO, Hand Exoskeleton for Rehabilitation Objective, to recover flexion and extension of the fingers in patients following stroke. This novel, low-cost exoskeleton is 3D-printed on textiles and controlled by motor imagery-based BMI [83].

Regarding prosthetic control, Gao et al. developed a motor imagery-based BCI to control a prosthetic leg walking in different terrains, intending to reproduce natural human gait [46]. Gannouni et al. proposed a BCI system that can detect finger movements [47]. Fuentes-Gonzalez et al. designed an EEG-controlled prosthetic arm [48], similarly to Zhan Hong et al. [70]. Murphy et al. presented a case study of BCI for controlling a prosthetic knee in transfemoral amputees with only the EEG signals arising from movement imagination [73].

Although less common, invasive EEG-based HMIs also exist. For instance, Li et al. tested the feasibility of an HMI based on the acquisition of sEEG signals to control a prosthetic hand [74]. Morishita et al. proposed an invasive BCI-based prosthetic arm. In particular, they decoded the motor intention by estimating muscle activity from ECoGs [76]. Yanagisawa et al. presented an invasive BCI system for controlling a prosthetic hand by using ECoG signals of a poststroke patient [78]. Finally, Fatima et al. reviewed intracortical BMIs for controlling upper-limb powered muscle and robotic systems in SCI patients [19].

Furthermore, these technologies can also be used as nonverbal communication tools. For example, Kashihara et al. proposed a BCI that allows interpretation of emotions by decoding facial expressions. In detail, the user is asked to look at some face images on a monitor. The visual stimulation triggers the activation of specific brain areas. Applying a classifier to the acquired EEG signal makes it possible to capture the subject’s reaction, which is correlated to his actual emotional state [84]. In addition, a BCI can be beneficial also in case of blindness, as described in [6]. Finally, a wide range of BMI-based neuroprostheses should be mentioned [23].

Moreover, it is noteworthy that another technique for BCI/BMI applications is functional Near-Infrared Spectroscopy (fNIRS), which acquires a biosignal that measures changes in blood oxygenation under the activation of specific brain areas. For example, this kind of BCI/BMIs can be used as a nonverbal communication tool or for controlling a wearable robotic exoskeleton [26,27].

Table 2 shows a summary of the selected EEG-based HMIs.

#### 3.1.2. EMG-Based HMIs

EMG reflects the electrical activity of skeletal muscles during voluntary contraction. The EMG signal is made of superpositions of small Motor Unit Action Potential waveforms with random phases. Hence, the EMG signal does not have a specific waveform but rather an interference pattern. Concise parameters of EMG are often used to monitor muscle contraction. The EMG time-varying RMS and the EMG linear envelope are largely used to assess the level of muscle contraction and the strength it develops (the stronger is the muscle contraction, the greater is the number and the frequency of active fibres, and, in turn, the higher is the amplitude of the EMG). Some other EMG parameters computed in the frequency domain can provide information about muscle fatigue. Since the EMG signal is specific to a single muscle, through the use of multiple electrodes, many muscles can be monitored simultaneously to decipher complex actions such as hand gestures or even movements of a single finger. EMG can be recorded noninvasively by employing skin-mounted electrodes (the so-called surface EMG (sEMG), which is by far the most used option) or invasively using implanted electrodes into the muscle (enabling more specific information on neural control) [44,60,86].

Currently, sEMG is the most widely used signal in biomedical HMI applications for prosthetic control [26,27,28,33]. It is easy to access and provides an intuitive control strategy to reproduce the function of a biological limb. This biopotential contains information about neural signals transmitted from the brain to muscles to perform a motor task. Hence, it allows for capturing the subject’s movement intention. EMG-controlled artificial limbs are referred to as myoelectric prostheses. Amputees represent the primary users of these assistive technologies. Indeed, people with a missing limb need to replace the lost function, in order not to suffer from their disability and to carry out daily activities autonomously and as naturally as possible. In the case of postamputation patients, the sEMG signal must be acquired from muscle groups of the residual limb, generally on the forearm or on the leg stump. Information associated with movements is captured from the EMG signal and converted into input commands to the prosthesis. To this aim, in recent years, machine learning algorithms, which substantially consist of feature extraction and classification steps, have gained ground in the biosignal analysis field. On this trend, Eisenberg et al. presented a novel segmentation technique that allows separating muscle contraction and rest periods of sEMG signals to perform real-time hand movements classification [53]. Tavakoli et al. proposed a simple, low-cost and efficient approach for hand gesture recognition from a single-channel sEMG. In particular, this method can recognize four hand gestures, hand closing, hand opening, wrist flexion, and double wrist flexion to control a prosthetic hand [54]. Bai et al. proposed a deep learning gesture recognition method from multichannel sEMG [87].

In contrast, Cao et al. presented a sEMG detection and action recognition system based on overlapping sliding window analysis and synchronous command transmission for real-time manipulator control [88]. Besides, Benatti et al. designed a wearable prosthetic hand controller based on gesture recognition that is able to communicate with a smartphone via Bluetooth, providing a customized control strategy [89]. In [90], an EMG-driven upper limb prosthesis for amputees living in developing countries was presented. The EMG signal is acquired via an Electro Resistive Band (ERB) sensor, which is low-cost, cheap, washable, and withstands wear and tear. The EMG signal’s amplitude drives the motor to set hand opening or closing commands. Polisiero et al. [91] designed a low-cost, EMG-controlled prosthetic hand intended for amputees living in developing countries. Two-channel sEMG signal is acquired on the forearm muscles and, after a preprocessing phase, the EMG envelope is computed to detect muscle contraction. Afterward, it is compared with a proper threshold to issue prosthesis hand opening or closing commands. In this way, the motor torque is proportional to EMG amplitude.

Gailey et al. provided a proof of concept of an online EMG-based prosthetic hand control method that can predict different hand gestures and individual finger forces via a Support Vector Machine (SVM) classifier [92]. Bernardino et al. proposed a control method for a 3D-printed prosthetic hand that uses the sEMG signals acquired from abdominal muscles [93]. In [94], pattern recognition from three-channel sEMG was performed to control a five-fingered underactuated prosthetic hand. The control method is divided into two main steps: (i) feature extraction through parametric autoregressive (AR) model, wavelet transform, and integral of EMG signals, and (ii) classification via a variable learning rate (VLR)-based, three-layer feedforward neural network applied to EMG features. It can discriminate between flexion and extension of three fingers: thumb, index, and middle. Carrozza et al. designed a novel EMG-controlled prosthetic hand that allows hand opening and closing tasks [95]. In [96], a novel sEMG sensor using polypyrrole-coated nonwoven fabric sheet was used to capture a subject’s motion intention for controlling a myoelectric prosthetic hand. Brunelli et al. proposed a fully wireless, low-cost, wearable multichannel sEMG acquisition system that can communicate via Bluetooth with a mobile processing device, which in turn can send motor commands to a prosthetic hand [97]. In [98], individual and combined finger movement features were extracted from sEMG signals using time–frequency distribution. The performances of three different classifiers in discriminating these movements were compared to obtain a more dexterous prosthetic hand control. Khushaba et al. presented a pattern recognition method that can classify different individuals and combined finger movements from two-channel sEMG to control a prosthetic hand [99]. In particular, a Bayesian data fusion postprocessing algorithm was applied to enhance the classification performance.

Furthermore, Kamavuako et al. [100] investigated the feasibility of using the intramuscular EMG (imEMG) signal for the control of a myoelectric prosthetic hand by applying a Fitts’ Law test, while Dewald et al. [101] presented a case study of imEMG-based prosthetic control. Al-Timemy et al. proposed a multichannel sEMG-based pattern recognition method to classify different individual and combined finger movements for dexterous hand prosthesis control [102]. In detail, they performed feature extraction through time domain–autoregressive approach, feature reduction by applying orthogonal fuzzy neighbourhood discriminant analysis (OFNDA), and classification via a Linear Discriminant Analysis (LDA). Zhang et al. designed an anthropomorphic EMG-controlled prosthetic hand [103]. Dalley et al. proposed a two-channel sEMG-based method for controlling a multigrasp prosthetic hand [104]. Russo et al. designed an artificial EMG-controlled prosthetic hand. In detail, sEMG signals are acquired via MyoWare muscle sensor, and three different hand movements are recognized through a SVM classifier [105]. In [106], neck and face sEMG signals were used to control a voice rehabilitative device for people with total laryngectomy.

Moreover, the “Myo armband” by Thalmic Lab is worth mentioning, a wearable device for human–machine interaction (discontinued from the market). It detects muscle activity via eight dry sEMG sensors and hand/forearm movements through a nine-axes Inertial Measurement Unit (IMU). The acquired data are transmitted via Bluetooth and can be used to control different devices (PC, robot, prosthetic hand, etc.), as described by Visconti et al. in [107]. Furthermore, Lu and Zhou proposed a hand-free HMI driven by facial EMG signal for controlling electronic devices (e.g., mouse, touch screen, etc.) [108].

In [109], sEMG signals were used to control an electric wheelchair for patients suffering from SCI at lower cervical levels. The classification performances of a SVM and a k-Nearest Neighbour (kNN) were compared to distinguish five different commands: left, right, forward, stop, and rest. Kalani et al. presented an sEMG-based teleoperated masticatory rehabilitation robot [110]. Briefly, three kinematic parameters are predicted using eight-channel sEMG (acquired from four jaw muscles of a teleoperator) and used to control the robot’s masticatory trajectory. Alibhai et al. performed gesture recognition from sEMG signals acquired via a Myo armband to control an intelligent wheelchair [39]. In [37], a three-channel sEMG signal was used to wirelessly control the commercially available JACO robotic arm. This control method was proved to be simple, low-cost, comfortable, easy to use and, above all, suitable for people with severe motor disabilities who cannot make use of the joystick. Song et al. addressed the problem of muscular fatigue by proposing a robust pattern recognition method based on a fuzzy neural network to control a powered wheelchair [40]. Laksono et al. investigated the best model to classify three different upper limb movements, i.e., elbow extension, shoulder extension, and combined elbow and shoulder extension, from a three-channel sEMG signal for the control of a robotic arm by applying machine learning [38]. In detail, they compared 48 different classification models that were obtained with or without data division by time, with or without Teager–Kaiser energy operator (TKEO), and by using conventional features in the feature extraction phase and testing eight different classifiers in the classification step. In [41], facial sEMG signals were used for hands-free control of an intelligent wheelchair. In particular, the muscle movements during chewing, namely single jaw click and double jaw click, are converted into commands to the device, i.e., forward, backward, left, right, and stop. In [111], facial sEMG signal was acquired during forehead single and double click movements and used to issue commands to an intelligent wheelchair (i.e., forward, backward, turn left, turn right, and stop). Hamedi et al. proposed an EMG-based facial expression recognition method suitable for HMI applications [112].

In the field of exoskeleton control, Wege and Zimmermann presented an EMG-controlled hand exoskeleton for rehabilitation purpose. Ten-channel sEMG is acquired on the forearm to detect subject’s motion intention. After blind source separation, rectification, and low pass filtering, the signal is decomposed into different components to capture each finger’s flexion and extension. Then, corresponding force values are computed and compared with appropriate thresholds to generate a trajectory executed by the motor control [113]. Ho et al. designed an EMG-driven exoskeleton for hand closing and opening rehabilitation in chronic patients after stroke. EMG sensors are placed on the forearm to capture voluntary contraction of abductor pollicis brevis and extensor digitorum. Hand closing is triggered by 20% exceeding the maximum voluntary contraction value of the former, whereas hand opening is associated with 20% exceeding the maximum voluntary contraction value of the latter [114]. Besides, Loconsole et al. proposed an EMG-controlled rehabilitation hand exoskeleton for bilateral grasp training. The signal is acquired on three forearm muscles of the healthy upper limb and used to control the grasp function of the impaired one [115]. Hussain et al. presented an EMG interface for the control of a supernumerary robotic finger. In particular, they used an EMG armband for hand gesture recognition to control motion and an sEMG channel to control joints compliance [116].

Furthermore, Abdallah et al. designed a 3D-printed EMG-driven robotic exoskeleton to assist finger flexion and extension movements in patients following stroke [117]. Secciani et al. presented a low-cost, fully wearable EMG-driven hand exoskeleton to assist impaired people in daily life activities [118]. In detail, sEMG signals are acquired on the extensor digitorum superficialis and flexor digitorum superficialis to capture the subject’s motor intention, i.e., hand opening, closing, or resting. Song et al. proposed an EMG-controlled exoskeleton for wrist flexion and extension rehabilitation in patients after stroke [119]. In [120], a myoelectric control method for a lightweight, 3D-printed upper arm exoskeleton, intended to improve muscle strength in home-based rehabilitation sessions, was presented. In detail, the method exploits sEMG signal acquisition through a Myo armband and different training load classifications via a kNN. Cai et al. presented ReRobot, an EMG-controlled upper limb exoskeleton for rehabilitation of poststroke patients by exploiting mirror therapy [121]. Indeed, sEMG signals acquired on the healthy side are classified by a SVM to decode the subject’s motor intention, then executed by the exoskeleton attached to the impaired side. Yin et al. extracted gait cycle durations (GCDs) from an eight-channel sEMG signal using the autocorrelation and the Bayesian fusion algorithm for controlling the motion speed of an exoskeleton–treadmill gait rehabilitation system [122]. In [123], an upper limb EMG-controlled power-assist exoskeleton intended for elbow rehabilitation was developed. Lu et al. proposed a four-channel sEMG-based real-time control for a rehabilitation hand exoskeleton [124]. In [125], a custom lower limb robotic exoskeleton control method was presented by estimating the active joint torque from the sEMG signal.

La Scaleia et al. showed a novel approach for associating the EMG signal from shoulder muscles with leg kinematics, which can be adopted to control an avatar walking in virtual reality or a robotic lower-limb exoskeleton [126]. Finally, Lyu et al. developed an EMG-controlled knee exoskeleton for home rehabilitation of poststroke subjects. In particular, the EMG signal is acquired via Myo thigh band, which is obtained by combining two Myo armbands. In addition, for greater involvement of patients, they are asked to perform training sessions in a visuomotor game context [127].

Table 3 presents a summary of the considered EMG-based HMIs.

#### 3.1.3. ENG-Based HMIs

ENG is the recording of electrical activity directly from a peripheral motor nerve. ENG is an invasive technique that involves electrodes implanted in the neural tissue. ENG provides precise and high-resolved information on a group of neurons or even on a single neuron activity. Differently, EEG can only record general activities of the brain, while sEMG provides aggregated information about neuromuscular control [44,60].

In the last decade, even ENG signal has captured attention in biomedical HMIs, principally for prosthetic hand control. However, in the literature, there are still few studies about ENG-based HMIs [24]. The central idea is to extract user’s motor intention by recording the electrical activity of peripheral nerves. Noce et al. proposed an ENG-based hand prosthesis control. In particular, they acquired neural signals from an amputee’s median and ulnar nerves and computed the ENG envelope. Afterward, by applying typical EMG pattern recognition techniques, they performed hand gesture recognition without needing a feature extraction phase. Furthermore, for comparison purposes, they applied the proposed method also to sEMG signal, thus confirming the validity of this novel approach [52]. Nguyen et al. presented a bioelectric neural interface for hand prosthesis control, which is integrated with implantable microelectrodes to record ENG signal from peripheral nerves in the residual limb, and deep-learning algorithm decode subject’s motor intention [55]. Finally, Noce et al. developed an ENG-based classification algorithm for discriminating two different grasp commands to control a prosthetic hand [59].

Table 4 shows a summary of the selected ENG-based HMIs.

#### 3.1.4. EOG-Based HMIs

EOG is the measurement of a steady potential generated by the eyeball’s movements due to separated positive and negative electric charges. Therefore, a current flows from the cornea to the retina. This biopotential can be recorded by placing surface electrodes on both sides of the eye, either horizontally or vertically. Indeed, when the gaze is straight ahead, the EOG signal is zero because of the eyeball’s symmetric horizontal position for the two electrodes. On the contrary, when the gaze is shifted to one side, a nonzero EOG signal is detectable. The same principle is applied to measurement in a vertical configuration. Therefore, there is a relationship between the horizontal or vertical gaze angle and the EOG amplitude [44,45,60].

Various biomedical HMIs exploit eye tracking from EOG signals, enabling disabled people to interact with the outside world through eye motions. EOG-based assistive technologies can be used as nonverbal communication tools or for controlling a robot, a prosthesis, a wheelchair, a device in a smart environment, the cursor on a computer screen, and so on. Golparvar and Yapici designed a graphene textile-based wearable assistive device that allows eye tracking from EOG for remote control objects [56]. Zhang et al. developed an EOG-based HMI for smart home environment control (e.g., TV control, air conditioner control, wheelchair control) in the case of SCI patients. In particular, a visual stimulus, in the form of flashing buttons on a Graphical User Interface (GUI), is presented to the subject. Each button corresponds to a specific command. The user selects the input command by blinking his eyes synchronously to the flashes. The acquired EOG signal is submitted to feature extraction and classification operations to decode the subject’s intention [42].

Similarly, Huang et al. presented an EOG-based HMI for wheelchair control [57]. Martínez-Cerveró et al. developed an open-source hardware/software platform for eye movements recognition from EOG signal. In detail, this system is able to classify four different eye movement directions and can be used in HMI applications for assisted communication in paralysed people [128]. Moreover, Perez Reynoso et al. proposed an EOG-based HMI for real-time trajectory tracking of a manipulator robot by using neural network modelling [129]. Again, Choudhari et al. presented an EOG-based HMI for wheelchair control by converting voluntary single, double, and triple eye blinks into control actions, i.e., forward, right turn, left turn, and stop [130].

In contrast, Heo et al. developed a headband-type forehead EOG measurement system for HMI applications, consisting of wireless wearable sensors for detecting six class-eye movements [131]. Guo et al. developed a wearable HMI based on single-channel EOG recorded with a patchable sensor [132]. Finally, Wu et al. designed a single-channel EOG-based HMI integrated with encoding/decoding paradigms of eye blinking and looking up to interpret the user’s intention [133].

Table 5 shows a summary of the selected ENG-based HMIs.

#### 3.1.5. Hybrid Biopotential-Based HMIs

Hybrid or multimodal biopotential-based HMI is the result of combining different types of bioelectric signals to increase their performance and reliability. Moreover, by exploiting the advantages of each category and associating many functions, these HMIs improve their effectiveness as assistive and rehabilitation technologies. Indeed, they allow people with disabilities to perform multiple tasks or implement better control. Furthermore, various combinations of biopotentials exist.

In the field of robotic control, Gordleeva et al. presented a real-time hybrid HMI for controlling a lower-limb exoskeleton. In particular, they combined a foot motor imagery-based BCI and multichannel EMG signals acquired from leg muscles to extract user’s motor intention, then converted them into input commands to the exoskeleton [51]. Ferreira et al. proposed a hybrid HMI based on EMG signal caused by eye blinks and EEG signal from the cerebral area responsible for processing the visual information to control a robotic wheelchair [134]. Zhang et al. developed a multimodal HMI combining EOG, EMG, and EEG signals to control a soft robot hand. In detail, this HMI exploits hand motor imagery from EEG, looking-left and looking-right eye movements recognition from EOG, and hand gesture recognition from EMG [135]. Huang et al. proposed a novel hybrid BCI that acquires EEG and EOG signals and translates them into commands for controlling an integrated wheelchair robotic arm system. By interacting with dedicated panels on a GUI, the user can control turning left/right of the wheelchair by performing hand motor imagery and issue other commands to the wheelchair and the robotic arm through eye blinking and eyebrow raising [136]. Finally, Ma et al. presented an EOG/EEG hybrid HMI for single and multiple robot control, which is based on four class-eye movements recognition from EOG and ERPs classification after the application of visual stimuli [12,137].

Regarding prosthetic control, Arrow et al. demonstrated the feasibility of using ERG signals for improved myoelectric hand prosthesis control. ERG is recording a transient potential that develops on the retina’s surface or the cornea in response to a light stimulus [44,61]. In particular, they used a threshold-based control strategy. Indeed, the number of naturally occurring neuronal action potential spike trains, generated by the retina after a visual stimulation, is counted and then compared with a predefined threshold to set the command to be sent to the prosthetic hand in order to improve the response time and the desired grip [58]. Rezazadeh et al. designed a coadaptive and affective HMI, which is able to assess the subject’s mental workload from EEG signal, for a better myoelectric forearm prosthesis control [138].

Furthermore, again Rezazadeh et al. proposed a multimodal HMI based on the acquisition of facial EEG, EOG, and EMG to perform face gesture recognition. In detail, features related to facial movements, eye movement direction, and mental states are extracted from fEMG, fEOG, and fEEG, respectively, thus enabling nonverbal communication [139]. Iáñez et al. presented a hybrid HMI that combines EEG and EOG signals to move a dot on a GUI [140]. Finally, Laport et al. compared two different HMI systems based on single-channel EEG and EOG to control smart home devices [141].

Table 6 shows a summary of the considered hybrid biopotential-based HMIs.

### 3.2. HMI Control Based on Muscle Mechanical Motion

Information about muscle mechanical motion is of great interest to many biomedical HMI applications, particularly for assistance and rehabilitation purposes, providing alternative strategies for controlling prostheses, robots, virtual reality, or even devices in smart environment. The term “muscle mechanical motion” refers to all mechanical events occurring during voluntary contraction, of which the EMG represents the electrical counterpart. Their monitoring allows the measurement of mechanical-induced muscle morphological changes. Indeed, it is well known that a contracting muscle changes its shape or dimensions (e.g., its cross-section), and small vibrations due to the progressive recruitment of motor units can be perceived (i.e., the mechanomyogram). Furthermore, muscle contraction also involves an increased blood afflux and displacement of muscle–tendon groups or even bones [4,143,144,145,146,147]. Therefore, these features reflect three main mechanical components: *gross motion* of specific muscle groups with associated muscle swelling; *muscle vibrations*; and *movement of musculotendinous groups*. To the aim of muscle contraction detection, multiple sensor technologies and different acquisition techniques have been proposed.

#### 3.2.1. Muscle Gross Motion-Based HMIs

As mentioned before, muscle contraction is generally associated with muscle volume, cross-sectional area, and stiffness changes. Therefore, muscle contraction detection can be performed by measuring specific muscle or muscle group changes due to the so-called muscle gross motion. To date, several noninvasive sensor technologies have been developed that measure different physical quantities, which exhibit a direct or indirect relationship with contractile force, such as force/pressure sensors and triboelectric sensors. They are placed on the skin in order to record mechanical signals from the underlying muscles.

First, force sensors are sensitive to the force/pressure exerted by muscle contraction. A wide range of force sensors is available, such as resistive, piezoresistive, pressure, piezoelectric, and even optical fibre sensors. Many of these enable the recording of force myography (FMG) signals. Force myography is a noninvasive technique that uses force sensing elements to detect “changes in stiffness of corresponding musculotendinous complex against a default state” [4], providing information about muscle contraction in the low-frequency range (i.e., <10 Hz) [148]. Force-sensitive resistors (FSRs) change their electrical resistance as a function of the applied force and are widely used in FMG recordings. Several studies show the potential of FMG as an alternative control strategy for HMI applications, particularly for upper-limb prosthetic control [9]. In this context, Sakr et al. demonstrated the feasibility of estimating hand isometric force/torque from FMG signals recorded via 60 FSRs, which were embedded into four bands and placed in different locations around the arm [149]. Again, Sakr et al. showed the possibility of predicting force in dynamic conditions by using FSRs worn around the arm [150]. Ahmadizadeh et al. explored the application of feature selection to three high-density FMG datasets in order to reduce its dimensionality and, at the same time, achieve the same performance but with lower cost and complexity [151]. Xiao et al. proposed a novel FMG system, consisting of a strap embedded with eight FSRs, to detect different forearm positions for controlling a custom-made forearm pronation/supination exoskeleton [152]. Ferigo et al. presented a case study of an FMG-controlled bionic hand prosthesis for a transradial amputee [153].

Furthermore, Esposito et al. developed a piezoresistive array armband for hand gesture recognition equipped with only three FSR-based sensors. It is able to detect eight different hand gestures and can be used for prosthetic control [154]. A study by Prakash et al. proposed an FMG-controlled prosthetic hand for upper limb amputees. An FMG sensor consisting of two FSRs was applied on the residual forearm of an amputee to detect muscle contractile force, then converted into input commands to the prosthesis [144]. Esposito et al. also presented an alternative approach to EMG, with comparable performances, for improved hand prosthesis control based on muscle contraction detection via an FSR-based sensor [143]. The sensor was placed on a forearm muscle, proving to be as effective as the EMG envelope to control a hand prosthesis prototype [155,156]. Ha et al. explored the prediction of hand gestures by applying piezoelectric sensors around the forearm to map muscle contraction [157,158]. A piezoelectric sensor converts its mechanical deformation due to the applied force into an electrical signal. Ahmadizadeh et al. showed the feasibility of using selected locations of FSRs for FMG-controlled prosthesis with performances comparable to high-density FMG [151].

Furthermore, Fujiwara et al. proposed a low-cost optical FMG sensor for hand gesture recognition based on modulation of light intensity due to the applied force by the microblading effect, which can be applied for hand prosthesis control [159]. Moreover, Bifulco et al. tested the feasibility of a conductive rubber sensor, that changes its electrical resistance when stretched, to detect muscle contraction on the forearm, and also to control a hand prosthesis prototype [160]. Radmand et al. explored high-density FMG for prosthetic control. An array with a high number of FSRs, mounted into a prosthetic socket, was used to measure changes in surface pressure on the forearm [161]. Again, Cho et al. investigated the feasibility of FMG-controlled upper extremity prostheses [162].

Muscle contraction detection can also be performed via triboelectric sensors, which convert their mechanical deformation into electrical output, thus acquiring contractile force. The working principle of such a sensor technology is based on electrification and electrostatic induction phenomena occurring when materials with different electronegativities come into contact. Dong et al. developed a wearable triboelectric HMI, in the form of a smart glove, using a nanophotonic readout for hand robotic control and virtual/augmented reality applications [163]. Similarly, Zhu et al. presented a smart glove equipped with elastomer-based triboelectric nanogenerators and piezoelectric mechanical stimulator for robotic control and virtual/augmented reality applications [16]. Finally, An et al. proposed a tattoo-like triboelectric self-powered wearable sensor for controlling robots or devices in a smart environment [164].

Moreover, some researchers proposed a novel, invasive approach for hand prosthesis control by tracking the position of permanent magnets directly implanted into the upper residual muscles of amputees. By externally measuring changes in the magnetic field due to magnets displacement, it is possible to detect the force exerted by muscle contraction and thus to capture valuable information to issue commands to the prosthesis. This innovative HMI was called myokinetic controller [165,166].

Table 7 shows a summary of the considered muscle gross motion-based HMIs.

#### 3.2.2. Muscle Vibrations-Based HMIs

During muscle contraction, muscle mechanical vibrations occur due to three main processes: (1) internal muscle vibrations, (2) oscillations of the human motor system (e.g., tremor and clonus), and (3) artifacts [172]. Mechanomyography (MMG) is a noninvasive technique that allows capturing high-frequency information (i.e., from 1–2 Hz to 100 Hz [3]) related to muscle vibrations. Islam et al. reported that MMG frequency content is closely related to the resonant frequency of muscle, which is affected by muscle stiffness [173]. However, the origin of the MMG signal is not yet fully understood. In Beck et al. [3], it is suggested that the MMG signal reflects three main physiological phenomena: (1) gross muscle movement during contraction, (2) muscle lateral oscillations at its resonant frequency, and (3) muscle fibres dimensional changes. Indeed, the recruitment of motor units during contraction results in dimensional changes of muscle fibres (e.g., their shortening and increase in diameter), which produce oscillations, i.e., pressure waves, that propagate from muscle up to the skin where they are detectable [174,175]. Different technological solutions have been proposed for MMG recordings, such as microphones, accelerometers, piezoelectric, or laser distance sensors [3,173,174,175]. Asheghabadi et al. presented a single-site MMG sensor consisting of a piezo plate and a strain gauge, which can capture both electrical and acoustic features from vibrations of a single muscle to perform multichannel finger pattern recognition [146]. Castillo et al. designed a wearable armband equipped with four MMG microphones and placed around the forearm to study the relationship between distributed normal force and MMG informational content. In detail, they showed that, as average force increases, tissue viscoelasticity changes, resulting in increased mechanical conductivity. In this way, the sensor is able to detect vibrations of deeper muscles with improved discriminative power, which is very useful in HMI applications for prosthetic control [176]. Moreover, Wicaksono et al. proposed a wireless synchronous carbon nanotube piezoresistive patch sensor to record MMG signal from leg muscles, which can be used for prosthetic or robotic control [177]. Finally, Xie et al. performed MMG recordings via two accelerometers for hand and wrist gesture recognition to perform multifunctional prosthetic control [178].

Table 8 shows a summary of the considered muscle vibrations-based HMIs.

#### 3.2.3. Muscle–Tendons Movement-Based HMIs

It is worth mentioning other acquisition techniques that detect movement of musculotendinous groups resulting in morphological changes. In this context, Wu et al. presented an HMI using Electrical Impedance Tomography (EIT) for hand prosthesis control. The system performs electrical bioimpedance imaging, thus capturing changes in electrical conductivity under the movement of muscles and bones. In particular, an array of bioimpedance electrodes in a wristband was placed around the forearm to recognize nine hand gestures [145]. Furthermore, there are several HMI applications based on ultrasounds (US). They are safe, provide high temporal/spatial resolution, and can be acquired noninvasively. However, the major drawback is represented by the cumbersome US probe. Huang et al. compared simultaneous EMG recording and US imaging acquired on the forearm in terms of finger gesture recognition accuracies. They concluded that the US allows more dexterous and accurate control, thus showing its feasibility for prosthetic or robotic control [179]. Li et al. proposed a multichannel HMI based on an armband with US transducers for finger gesture recognition. It is designed to be applied in rehabilitation robotics [180]. Furthermore, Ortenzi et al. presented a comparative study of features and classification methods in US-based HMI for hand prosthetic control [181]. Sikdar et al. developed a novel method for predicting dexterous individual finger movements by imaging muscle activity using a wearable ultrasonic system [182].

Moreover, Sierra González and Castellini tested the feasibility of US-based HMI in a realistic scenario. They showed a linear relationship between the spatial first-order US features of the forearm and hand kinematic. In this way, it is possible to predict forces at the fingertips, thus revealing very useful for controlling a prosthesis [183,184]. Finally, two studies presented HMIs based on Sonomyography (SMG), a novel technique based on US acquisition that is able to detect muscle architectural changes and can be used to control a hand prosthesis. In particular, they investigated changes in muscle thickness during wrist flexion/extension. Maximum values of SMG are associated with wrist extension and, therefore, fingers opening, whereas minimum values of SMG imply wrist flexion and, thus, fingers closing [147,185].

Table 9 shows a summary of the considered HMIs based on muscle–tendon movements.

#### 3.2.4. Hybrid Muscle Mechanical Motion-Based HMIs

Finally, HMIs combining different techniques of muscle mechanical motion detection also exist. Esposito et al. presented a piezoresistive FSR sensor that is able to measure changes in muscle cross-sectional area and MMG signal, proving very useful for prosthetic control [143]. Booth and Goldsmith developed a wrist-worn piezoelectric sensor for finger gesture recognition. The sensor is able to capture both MMG signal and changes in muscle shape, providing information that can be used for prosthetic, robotic, virtual reality, or smart environment control [186].

Table 10 shows a summary of the selected hybrid muscle mechanical motion-based HMIs.

### 3.3. Body Motion-Based HMIs

#### 3.3.1. Image-Based Body Motion HMIs

A further class of HMIs is aimed at tracking the motion of different body parts, such as the eyes [43,187,188], the upper and lower limbs [189], and the head [190,191,192], via different kinds of vision devices. Eye-tracking technologies are usually based on infrared (IR) illuminators pointed at the eyes. IR sensors or cameras capture the reflected IR light to determine the gazing point on a screen and control a cursor to interact with various applications. The other vision-based HMIs mainly rely on standard cameras and sometimes also on depth cameras and involve a processing stage to recognize the gestures performed by the tracked body parts, e.g., translations and rotations of the head and various kinds of hand gestures. Moreover, a branch of vision-based HMIs comprises devices for exergames, which require active body movements to control the gaming experience and have potential applications for disease prevention, health promotion, and rehabilitation [18,20,29,30,31,32].

Maule et al. proposed an eye-tracking-based HMI called RoboEye [187]. RoboEye is composed of a standard power wheelchair integrated with an innovative, cost-effective, and user-friendly control system based on an eye-tracking system, a 3D camera, and a computer monitor. The system provides the users with comfortable navigation and two driving options, namely “direct” and “semiautonomous”, which allow them to move easily and autonomously within their homes. The natural modality uses eye-tracking to detect gazing at different areas of the monitor and provide continuous control of frontal and angular wheelchair velocities. This modality also enables efficient control of the wheelchair for users who cannot use standard interfaces (e.g., joystick, keyboard). The semiautonomous modality allows navigation toward a selected point in the environment by just pointing and activating the wished destination. At the same time, the system autonomously plans and follows the trajectory that brings the wheelchair to that point with the support of the 3D camera.

Bissoli et al. presented an intelligent home system that provides eye-tracking-based control of four devices, namely a television, a radio, a lamp, and a fan, as well as remote monitoring via the Internet of Things (IoT) protocols [43]. The control system and remote monitoring are intended for users with severe disabilities and related caregivers. The eye-tracking interface replaces the mouse control of a personal computer (PC): the changes of gazing points are translated into mouse movements, while the persistence in the same point for few seconds is translated into a mouse click. The system has been first tested on 29 healthy participants and then on a woman with severe disabilities in her own home for seven days. The efficacy of the smart home system was evaluated via a System Usability Scale (SUS) questionnaire, which was administered to both the healthy subjects and the person with severe disabilities, reporting very high scores.

Lin et al. proposed an HMI to control a computer mouse [188]. An IR camera captures images of the user’s eye, illuminated by an auxiliary infrared LED source. Features of the pupil are extracted to determine the gazing point on the screen, thus allowing the user to control a PC mouse. An autocorrection method is implemented to correct the gazing point estimate, leading to improved eye-tracking-based mouse control in the 90% of involved subjects, who achieved an overall accuracy of 97%.

An HMI for smart home control based on a Virtual Interactive Blackboard (VIB) has been designed by Conci et al. to remove any physical connection between the user and the domotic system [189]. VIB is based on the low-cost hardware architecture: the visual interface is projected through a beamer on a flat surface. A fixed webcam achieves the user’s gesture, which captures the scene at 10 fps. To achieve real-time processing, unnecessary information is first removed. The illumination is assumed to be slowly varying, so a background suppression procedure is performed by discarding illumination variations and focusing on areas corresponding to human skin colour to simplify hand tracking. An AdaBoost classifier is trained to provide real-time hand gesture recognition. Three hand gestures, namely opening, closing, and finger-pointing, are used to control the position of a cursor, trigger clicking actions, and trace curves for writing and drawing. A central processing unit connects the VIB interface to actuators taking charge of user commands, which can access several services (e.g., TV, phone) and control the related parameters (tuning, switching, opening–closing, moving).

A HMI based on head and mouth movement analysis has been proposed by Baklouti et al. as a control system for an exoskeleton, designed to compensate for the loss of upper limbs mobility in patients suffering from myopathy [190]. The system is based on a monocular camera to capture the user’s head and provides two control modalities, namely head control and mouth control. The head control uses an attention region approach to minimize the computational burden of face detection, then performs, in real time, a face detection and global feature extraction via Adaboost, and finally a pose estimation that provides the translation and rotation matrix of the head, which is used to generate an exoskeleton command. The mouth control performs a mouth extraction by combining a colorimetric method (thresholding on Q component of the YIQ colour space) with edge detection and active contour to locate extremum points for mean square fitting of lips profile with second-order polynomials. It then classifies the mouth expression from the modelled lips profiles to generate a command for the exoskeleton.

Chang et al. proposed an HMI for people who cannot use their hands to support using a computer or speaking [191]. A camera captures images of user’s head movements. An initial skin colour adjustment is performed to optimize the tracking of the user’s face within the frames. A binary mask is obtained with a group of pixels that roughly covers the user’s face in each frame. The barycentre of this group of pixels is computed to determine head movement direction among the eight directions considered by the system (two along the vertical axis, two along the horizontal axis, and two along each of the two 45° diagonals). The head tracking system is used to compose two-digit or three-digit codes corresponding to letters or sentences in predefined tables, which are then reproduced via a sound-generating system to allow disabled users to communicate.

A HMI based on head movement recognition has been presented by Gautam et al. to control a small robotic car [192]. A camera captures the user’s head images, which are processed to extract binary maps of face pixels. The image field is divided into three areas: left, centre, and right, and the head movement is classified based on the area of the binary map, including the most significant number of face pixels. The result of the classification is translated into a command for the robotic car, namely “turn left”, “go ahead”, and “turn right”. When user’s head falls out of the image field, i.e., no face pixels are detected in all areas, a “stop” command is sent to the robotic car.

Furthermore, in the context of the vision-based HMIs, it is worth highlighting the impact of the “exergames” on physical and cognitive functions, as demonstrated by different studies published over the last decade. Rosly et al. [31] presented a review on exergaming applications for subjects with neurological disabilities. They underlined that exergaming can provide outcomes with equivalent dose-potency as traditional physical exercise in clinic or home environments. Another survey by Reis et al. [32], showed that exergames could be used as a complement to traditional forms of motor rehabilitation in older subjects, improving their balance, gait, muscle strength, upper limb function, and dexterity. A combined intervention in which traditional physiotherapy is integrated with exergames appears to be more efficient than each type separately. Exergames were also used as a rehabilitation treatment for people affected by Parkinson’s disease [20] and MS [18]. Furthermore, the commercial device “Microsoft Kinect” (an RGB–D camera) was widely tested for motor rehabilitation purposes, as reported in the review by De Gama et al. [29]: many studies presented Kinect-based systems in which the subject had to simulate in a virtual environment a functional activity and achieve some objects to complete a task. A Cochrane review by Laver et al. [30] found evidence that virtual reality and interactive video gaming for stroke rehabilitation may be beneficial in improving global motor, upper limb function, and activities of daily living (ADL) when used as an addition to usual care or when compared with the same dose of conventional therapy. In summary, these reviews indicate that exergames appear to be a viable and effective rehabilitation tool for people with neuromuscular diseases.

Below are some articles that feature different technologies for exergaming purposes. A mapping study by Gmez-Portes et al. [193] showed how exergames could address home rehabilitation for children and teenagers. In particular, the study presents a home rehabilitation software prototype based on “Microsoft Azure Kinect DK”. A virtual avatar, showed in a connected screen, mimics the patient’s movements, and it is necessary to perform certain tasks to achieve the predetermined goals. Palaniappan et al. [194] tested the “HTC Vive” for virtual reality exergames on patients affected by spinal cord injury. The authors used the “Vive tracker” to track the movements of the patient’s arm by attaching it to a Velcro hand strap through a 3D-printed interface plate (the standard controller requires fine motor control to grip and use buttons). A further study by Nguyen et al. [195] showed an exergames room based on the “Jintronix Rehabilitation System (JRB)” (a virtual reality software compatible with the Microsoft Kinect) and the “Meditouch HandTutor (MHT)” (a set of electro goniometers measuring wrist and elbow movements) to interface the patient in stroke rehabilitation with the virtual reality and guide him in performing specific movements.

Table 11 outlines the image-based body motion HMIs studies included in the current survey.

#### 3.3.2. Nonimage-Based Body Motion HMIs

Body motions, such as hand or finger motions, are generally tracked by vision-based systems. An alternative approach is based on wearable devices, such as inertial sensors (the most widely employed for human motion recognition), touch sensors, strain gauges, flex sensors, and ultrasonic sensors. Some studies based on these technologies with assistive and rehabilitative purposes are reported below.

A study by Chuang et al. [196] focused on a smart glove equipped with flex sensors (variable resistors with the degree of deflection) for finger gesture recognition purpose. The study aimed to detect different thumb, index finger, and middle finger movements using neural network algorithms. A further paper by Dong et al. [197] proposed a smart glove embedded with piezoresistive strain sensors based on stretchable polydimethylsiloxane–carbon black to recognize finger gestures and control robot fingers. Zhu et al. [198] presented a different smart glove based on stretchable conductive metal-coated yarns (a nylon yarn covered by a metal film) to remotely control a robotic hand and manipulate the colour switching of light by using gesture recognition. Hang et al. [199] detected various human body motions using a poly(acrylamide) hydrogel-based strain sensor. Thanks to the high extensibility of these sensors, the authors measured the bending of fingers, wrist, elbow, and knee and the cheek bulging. Gesture recognition and a robotic hand’s gesture control were experimented with by embedding five hydrogel sensors in a glove.

In the context of exoskeleton control, Ueki et al. developed a hand exoskeleton for self-controlled rehabilitation therapy. It uses a closed-loop master and slave system to assist flexion/extension, abduction/adduction of hand joints, and thumb opposability. In detail, the subject performs movements in virtual reality with his healthy hand wearing a glove (i.e., master level), which is equipped with 3D motion sensor. The movement is reproduced by the exoskeleton attached to the impaired hand (i.e., slave level), which uses three-axis force sensors [199]. Similarly, a further study by Rahman and Al-Jumaily [200] proposed a hand rehabilitation exoskeleton consisting of a master and slave system. The healthy hand wears the glove acting as master and is equipped with flex sensors, whereas the impaired one wears the exoskeleton acting as a slave. The glove wirelessly transmits data to the exoskeleton that executes commands for fingers flexion and extension. Again, Cortese et al. proposed a hand rehabilitation exoskeleton in the form of a master and slave system. The therapist wears a glove (i.e., master unit), while the patient wears the exoskeleton (i.e., slave unit). These two devices exchange data between them. In particular, the therapist guides the exercises to be performed by the patient. The glove presents six three-axis MEMS accelerometers, which obtain position vectors classified and compared with proper thresholds to understand the specific command (i.e., rest, grasp, pinch) and, eventually, its percentage [201].

Moreover, some approaches use only wearable inertial sensors. An example is the research by Han and Yoon [202], who experimented with a three-axis gyroscope fixed on the hand back intending to discriminate six hand gestures (up, down, left, right, clockwise rotation, anticlockwise rotation) and track the hand trajectories. Then the HMI was tested as a PC input device for controlling various applications (presentation, video player, web browser).

Table 12 presents a summary of the considered non-image-based body motion HMIs.

### 3.4. Hybrid HMIs

Hybrid HMIs are characterized by two or more technologies that work together to achieve a common goal [11]. Various combinations of biopotentials with vision systems, force sensors, accelerometers, US probes, microphones, IMU sensors, etc. have been presented in the last two decades. Compared with single modality HMIs, multimodality HMIs could enrich the controllability of the interface, optimize the communication methods, and improve the overall performance of the interface [204]. Many studies combined biopotentials with morphological sensors (force sensors, US probes, etc.) to obtain electrophysiological and morphological information of the same muscle simultaneously [13].

#### 3.4.1. Biopotentials and Image-Based Systems

The current subsection shows biomedical HMIs with hybrid controls based on biopotentials detection and vision systems. A study by Wei et al. [204] presented a hybrid HMI designed for hands-free control of an electric-powered wheelchair. Forehead EMG signals and colour face image information were used to identify movements of the human face. In detail, five winking and jaw clenching movement patterns were selected and classified, mapping into six control commands to drive an electric-powered wheelchair in an indoor environment.

Haung et al. [205] presented a multimodal emotion recognition system by combining facial images and EEG. The acquired images were classified by a convolutional neural network (CNN) architecture for facial expression detection. Different SVM classifiers classified the EEG signals. Finally, the facial expressions and EEG signals were combined to recognize the facial emotions.

Downey et al. [206] experimented with a combined control system for a robotic arm/hand on patients with spinal cord injury. Microelectrodes arrays were implanted on the motor cortex of the patients for realizing a BMI. Moreover, a computer vision system composed of an RGB–Depth camera was mounted above the arm base to identify objects by matching depth image templates from a model library. During the experiment, the subjects controlled the robotic arm/hand to perform grasping tasks.

A conference paper by Bu et al. [207] proposed a hybrid control method for prosthetic hand, combining EMG signals with a vision-based object classifier. Information of target objects (shape, dimension) are obtained from images and then utilized to generate control commands for motors in combination with the EMG signals.

A study by Malechka et al. [208] presented the “sBCI eye tracking system” composed of a headset that integrates multichannel EEG equipment, an eye tracker system (two cameras for tracking of left and right eye) based on video-oculography (VOG), an environmental observation camera, and an integrated visual stimulator for a SSVEPs. The system detects the user’s intention to interact with a specific device in its environment. McMullen et al. [209] presented a hybrid HMI named “HARMONIE system”. This system utilizes ECoG and depth electrodes within the motor cortex for realizing an iEEG-BMI, with the aim to identify the movement intention. A Microsoft Kinect sensor is used to record both the depth information and RGB of the experimental workspace. The patient’s gaze is captured by a monitor-mounted eye-tracking system. A patient implanted with iEEG electrodes can use the eye tracking system to visually select an object displayed in real-time on the monitor and provide control signals to an upper prosthetic limb.

Frisoli et al. proposed architecture for controlling a rehabilitation arm exoskeleton in reaching and grasping functions. In summary, the system is composed of a motor imagery-based BCI to decode the subject’s motion intention, an eye-tracking to detect subject’s gaze direction while selecting the target object in the task space, and a Kinect sensor to track the object in the 3D space and communicate its position to the robotic device [210].

Table 13 outlines the hybrid controls for HMIs based on biopotentials and image-based systems included in the current survey.

#### 3.4.2. Biopotentials and Mechanical Motion Detection

This subsection shows some biomedical HMIs with hybrid controls based on biopotentials and mechanical motion detection.

The human anatomy inspires a prosthetic hand presented by Dunai et al. [211]. It works by using sEMG sensors to allow the user to activate the prosthesis and FSR sensors to simulate the touch pressure of the fingers. The grasping is completed when the FSR in the thumb signal is above a given threshold.

Krasoulis et al. [211] proposed a multimodal control for prosthetic hand, which combines sEMG sensors with IMU systems. In detail, each EMG electrode incorporated a nine-degree-of-freedom (DOF) IMU, i.e., a triaxial accelerometer; gyroscope; and magnetometer measuring acceleration, angular velocity, and magnetic field, respectively. A total of 12 hybrid sensors were used for monitoring the activation of the arm and forearm muscles and provided control signals to the prosthetic hand. A further example of a hybrid sEMG-IMU interface is the study presented by Shahzad et al. [212] to evaluate the variation of the sEMG signals with the arm position. The system consists of multiple sEMG sensors and nine-DOF IMUs to measure the electrical activity of the muscles and the position of the forearm relative to the shoulder while performing six classes of hand motions. A conference paper by Kyranou et al. [213] also experimented with a hybrid control for robotic prosthetic hand, based on 12 sEMG sensors, each integrating a nine-DOF IMU. A multiclass LDA classifier was used to discriminate six hand grip patterns and use the predictions to control a robotic prosthetic hand in real-time.

Moreover, Jaquier et al. [214] experimented with a hybrid device composed of sEMG and pressure sensors to predict the activation of the wrist, hand, and single-finger for future prosthetic implementations. A proximal forearm device is a tactile bracelet with 10 pressure sensors (made by resistive elastomers); a distal forearm device is composed of 10 sEMG electrodes (Ottobock Myobock).

A different combined control for upper-limb prosthesis, presented by Guo et al. [215], was based on sEMG and near-infrared spectroscopy (NIRS) sensors (light sources at three wavelength: 730, 805, and 850 nm). The study investigated the classification accuracy in discriminating 13 wrist and hand motions. It tested the performance in controlling a virtual prosthetic hand by using four hybrid sEMG/NIRS to the forearm muscles of amputees.

A further type of hybrid control is the combined sensor sEMG/US presented by Xia et al. [13]. The paper proposed a portable hybrid sensor system: the sEMG electrodes and an US transducer (for A-Mode signals) are fixed into a module to detect electrophysiological and morphological information of the same muscle. An armband equipped with four hybrid modules is mounted on the subject’s forearm to recognize 20 different gestures (6 wrist motions, 13 finger motions, and a rest state motion).

Furthermore, Dwivedi et al. proposed a soft assistive exoskeleton that combines a glove and a muscle–machine interface in the form of a sensorised sleeve. The interface is equipped with EMG and FMG sensors to decode the subject’s motion intention. The acquired data are classified into different grasp types and used to trigger the motors [216].

Table 14 summarizes the selected hybrid controls for HMIs based on biopotentials and mechanical motion detection.

#### 3.4.3. Other Various Hybrid Controls

The following subsection shows some examples of biomedical HMIs not classifiable in the previous subsections whose controls are based on hybrid combinations of sensors (biopotentials, IMU, US, vision system, voice recognition, microphones, Radio-Frequency Identification (RFID), etc.) and whose application fields are principally involved in assistance or rehabilitation.

Ubeda et al. [11] presented a shared control combining a BMI with RFID technology to control a robot arm. RFID tags were placed in the experimental setup to give information about the position of the objects in the scene. An antenna, placed on the robot arm’s end effector, is used to read/write the tags. Information about objects’ positions is stored in the tags. The shared control system uses this information and supports the user to make high-level BMI decisions via mental tasks (move left, move right, pick, or place). The authors showed that the total amount of BMI commands can be significantly reduced thanks to the RFID tags.

A further study by Perez et al. [218] proposed a hybrid control for a robotic wheelchair to be used by people with severe motor disability. The system is based on a Visual-Based Interface (VBI) and an IMU sensor and combines the information of the user’s head orientation (obtained by both the VBI and the IMU sensor) for providing control signals to the wheelchair. In [219], another hybrid HMI for controlling a robotic wheelchair was presented, which combines six different control strategies, i.e., eye blinks from sEMG, eyes and face movements acquired using video cameras, head movements detected via an IMU sensor, sip-and-puff captured by a pressure sensor, and brain waves from EEG.

Voice recognition systems have also been experimented with for controlling wheelchairs. An example is the study by Anwer et al. [220] that presented an eye- and voice-controlled wheelchair. The device carries out both voice commands (on, right, left, stop), which are processed through an Arduino platform, and commands given by the movement of the eyes (to the right or left), captured with a front camera and processed with a Raspberry PI platform.

Another low-cost multimodal sensor was presented by Gardner et al. [221]. It consists of an acoustic MMG sensor, a nine-DOF IMU sensor, and a video camera. On an amputee participant, a compression sleeve containing both the MMG and the IMU sensors were positioned on the biceps. The participant wore the video camera on a pair of glasses. The study predicts different grasp strategies and the successive control of a commercial prosthetic hand (BeBionic Hand).

Moreover, in order to allow people with severe disabilities (ALS, CP, stroke, etc.) to communicate, a study presented a wireless home assistive system [222], consisting of various types of sensors: EOG (for detecting eyes movements) and switches (based on: push button, InfraRed, mercury, long–short tone, and pacifier) were used for generating a Morse Code. Afterward, a fuzzy algorithm-based-Morse Code recognition allowed it to provide input commands to a connected PC.

Table 15 presents a summary of the considered hybrid controls for HMIs based on various combinations of sensors.

## 4. Discussion

This survey analysed the literature on biosignal-based HMIs that use biopotentials, mechanical muscle movements, body motion, or their combinations as control signals, with applications in the field of assistance and/or rehabilitation. Journal and conference papers presented in the past 20 years were considered.

### 4.1. Statistical Analysis

**Figure 2:**
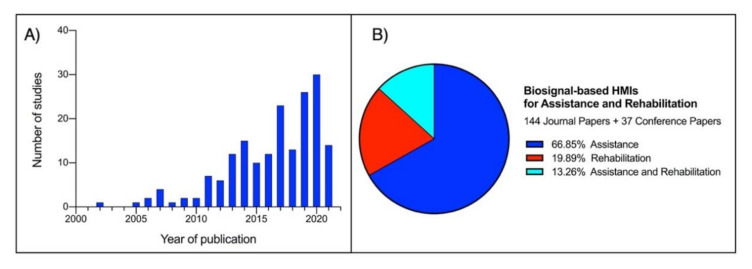
(**A**) Histogram illustrating the trend in the last two decades in the number of studies selected for the current survey. (**B**) Pie chart presenting the percentage distribution of the selected studies, according to the field of application.

Statistical data extracted from the selected papers (144 journals papers and 37 conference papers) are reported below. Figure 2 shows the number of publications over the last years: the trend is growing. This proves the ever-growing interest to develop HMI technologies for assistive and/or rehabilitative purposes. Figure 2B shows how the studies are related to the considered applications. The highest percentage (about 67%) pertains to the assistive field, while rehabilitation and assistance rehabilitation represent about the 20% and 13% of all studies, respectively.

**Figure 3:**
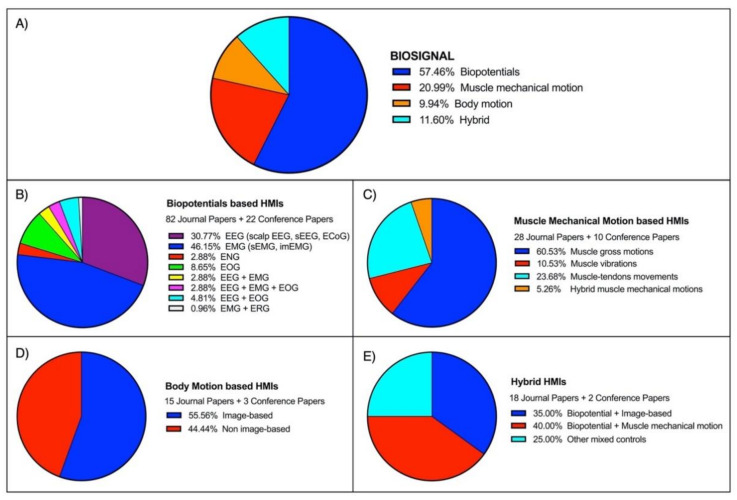
(**A**) Pie chart showing the percentage distribution of the biosignals implemented by the HMIs considered in this survey. (**B**–**E**) Pie charts representing the percentage distributions of the biosignals subcategories: (**B**) Biopotentials-based HMIs; (**C**) Muscle mechanical motion-based HMIs; (**D**) Body motion-based HMIs; (**E**) Hybrid HMIs.

Figure 3A shows the percentages of use of the biosignals for HMI control. Biopotentials constitute the highest percentage (about 57%), while muscle mechanical motion represents 21%. Lower percentages are associated with body motion controls (about 10%) and hybrid ones (about 12%). The pie charts in Figure 3B–E represent the distributions of the subcategories of biosignals. The most used biopotentials are still the EMG and the EEG (>75%). Among the muscle mechanical motion signals, the muscle gross motions are the most used ones and, together with the related tendon motions, are involved in about 85% of the studies. The studies on hybrid HMIs are well-balanced; however, it is worth highlighting that biopotentials are involved in 75% of these studies.

**Figure 4:**
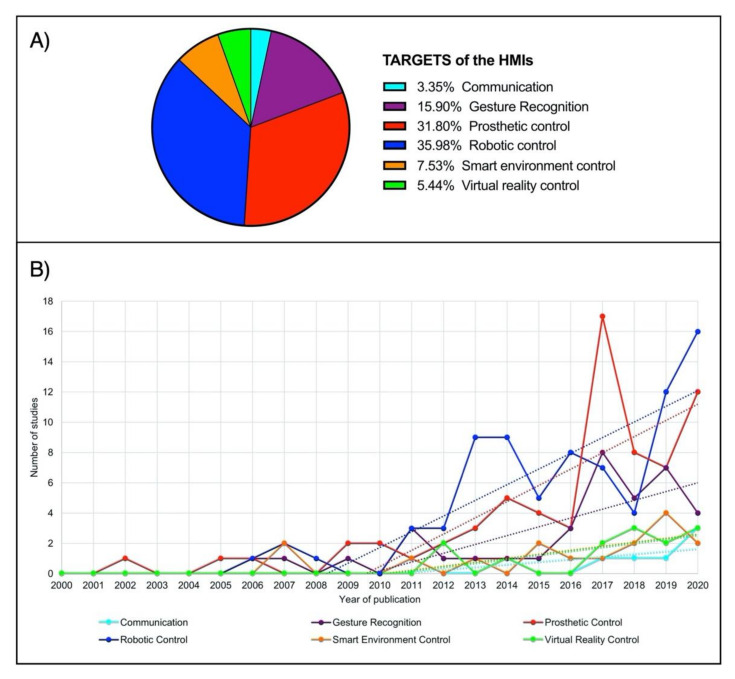
(**A**) Pie diagram illustrating the percentage distribution of the considered targets by the selected HMIs. (**B**) Graph illustrating the trend in the last two decades of each target (depicted in a different colour) of the considered HMIs with assistive and/or rehabilitative applications. Linear regressions (starting from 2010) are also superimposed as dashed lines.

Figure 4A shows the distribution of studies focusing on the different targets, while Figure 4B shows their timeline. About 70% of these studies addressed robotic and prosthetic control applications. The number of studies published before 2010 only represents about 6% of the total. Starting from 2010, a moderate increase can be observed in studies focusing on robotic control, prosthetic control, and gesture recognition, while studies on the other targets experienced only a minor increase.

### 4.2. Advantages and Disadvantages of Biosignal Categories

**Figure 5:**
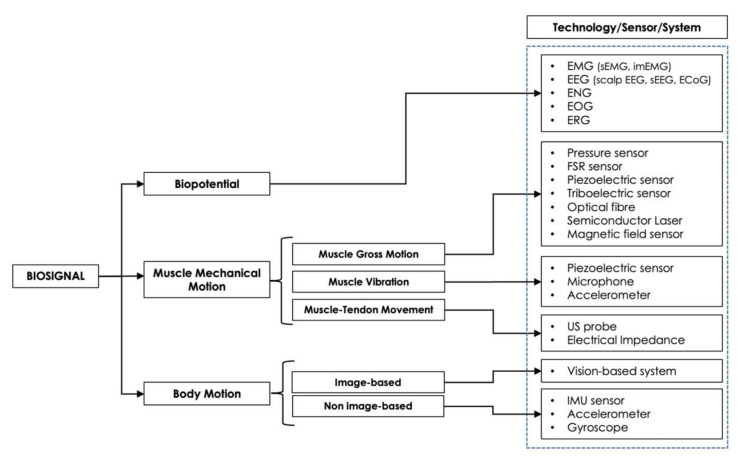
Types of biosignal and main technologies/sensors/systems used by assistive and/or rehabilitative HMIs.

A summary of the principal technologies/sensors/systems associated with each different macrocategory of biosignal is displayed in Figure 5. For the sake of simplicity, hybrid systems have been omitted.

It is interesting to briefly report the pros and cons of each macrocategory of biosignal-based HMIs.

Biopotential-based HMIs allow capturing the subject’s intention from different body regions (i.e., brain, muscles, peripheral nerves, or eyes). They are widely used for controlling all kinds of assistive and rehabilitation devices. BCI systems are promising technologies, particularly for people with severe motor disabilities, such as quadriplegia or paraplegia. Most biopotentials are acquired noninvasively (e.g., scalp EEG, sEMG, EOG). In this case, stable electrode placement and skin preparation are required. Wet electrodes need the application of a conductive gel to ensure better contact; on the contrary, dry electrodes are very useful for long-term recordings [47]. Amplification and real-time processing are needed because biosignals have small amplitude and are affected by noise, such as electromagnetic interference, motion artifacts, and crosstalk with other biosignals, and modifications over time (e.g., EMG is susceptible to muscle fatigue). Increasing use of machine learning techniques has been documented to decode the subject’s intention. Some acquisition techniques are invasive (e.g., ECoG electrodes are surgically implanted and may involve a risk of infection) and therefore more complex and expensive [65]. However, they allow acquiring signals from otherwise inaccessible regions with much higher specificity (e.g., ENG allows more intuitive prosthetic control). The use of this type of HMI is not intuitive. Therefore subjects must first be trained [52].

Muscle mechanical motion-based HMIs exploit the morphological changes of muscles during contraction. The FMG technique (force, pressure, piezoelectric, triboelectric sensors, etc.) detects muscle gross variation, allows performance comparable to EMG without using electrodes (no electrical risk), requires much simpler processing, has a lower cost and is less affected by electromagnetic and environmental interferences. On the other hand, FMG is susceptible to muscle fatigue and crosstalk of adjacent muscles and requires a suitable capacity for muscle contraction (technique not usable in subjects with severe motor disabilities) [4]. The MMG technique (microphone, accelerometer, piezoelectric sensor, etc.), which allows detection of muscle vibrations during contraction, has the advantage of being more sensitive than FMG in interpreting movement intentions, even in subjects with poor muscle contraction ability but is more prone to noise and motion artifacts [3]. As for the detection of muscle–tendon movements, the US technique guarantees good resolution for both superficial and deep muscles, but the US probe is bulky and expensive; it is not wearable and portable, so it is suitable only for clinical or research environments [183].

Finally, concerning the body motion-based HMIs, the image-based ones have the advantage of being contactless and easy to use in a home environment. They are able to detect the movement of body parts (e.g., head, hands, eyes) but are susceptible to illumination conditions, camera field of view, and overlapping objects. They require complex image processing algorithms to extract useful information for controlling devices, and real-time implementation is difficult. This type of interface is essentially used for smart environment and virtual reality control [30]. On the other hand, nonimage-based HMIs exploits wearable, low-cost sensors which can be embedded into electronic devices, e.g., smartphone, or in gloves that can turn out to be uncomfortable for the user [197]. They are mainly used for robotic control.

### 4.3. Latest Trends

**Figure 6:**
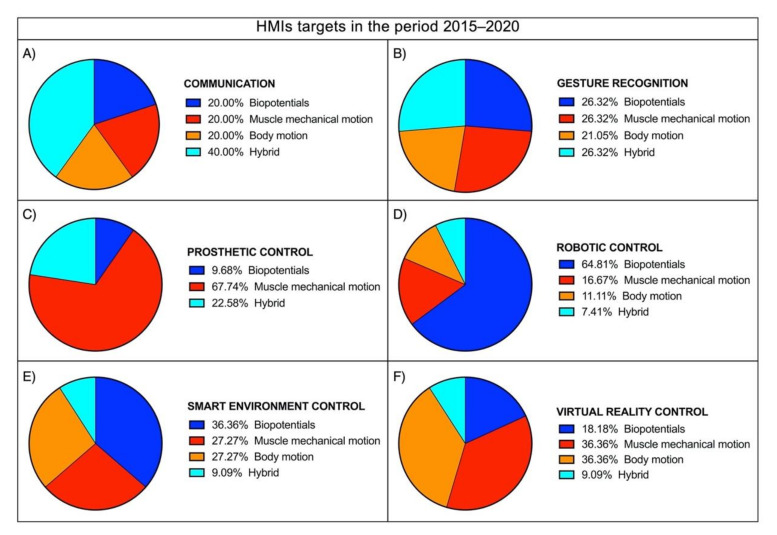
Pie charts illustrating the percentage distributions of the HMIs targets in the period 2015–2020: (**A**) Communication; (**B**) Gesture recognition; (**C**) Prosthetic control; (**D**) Robotic control; (**E**) Smart environment control; (**F**) Virtual reality control.

The pie charts in Figure 6 outline the percentages of use of the considered biosignals in each specific target’s state-of-the-art by considering the studies published only in the last five years (i.e., 2015–2020).

As opposed to the beginning of the 20 years considered in this survey, biopotentials are no longer the leading control signals of biosignal-based HMIs. Indeed, the use of muscle mechanical motion signals has reached or overcome biopotentials in all targets (more than 20% of studies), except for the robotic control, where biopotentials are still used in about the 65% of studies. In prosthetic control, muscle mechanical motion signals have been used in about 68% of studies, so their use has primarily overcome biopotentials (about 10% of the studies). In particular, the measurement of muscle gross motion (about 61% of all muscle mechanical motion signals) has experienced impressive growth as an alternative to EMG. Undoubtedly, FMG overcomes many well-known EMG limitations (e.g., artifacts due to unstable electrical contact, drying of pregelled electrodes over long periods, susceptibility to electromagnetic interferences), thus standing as a good, robust control signal for long-term HMI applications. On the other hand, while reliable standards are available for EMG measurement (e.g., SENIAM project), the properties of FMG sensors as well as their number and positioning for accurate measurements of muscle mechanical motion have not been standardized yet. Future research on FMG should address this issue to provide clear guidelines that ensure good quality measurements and increase the transferability of results obtained in different studies. Obviously, HMIs based on muscle mechanical motion are unfeasible for subjects with limited to no muscle activity, while those based on brain-related biopotentials, such as the BCIs, enable these impaired subjects to interact with machines that provide them with assistance or support their rehabilitation.

Body motion is prevalent in HMIs dedicated to controlling virtual realities and smart environments, as it has been used in about 30% of studies focusing on these targets. It has also been used in more than 20% of studies on communication and gesture recognition, and in few studies focusing on robotic control, its use is still limited. However, it has never been used for prosthetic control HMIs, probably because of the unsuitability of body motion capture technologies in the typical prosthetic control scenarios. Indeed, a great part of these technologies are image-based, so they do not fit very well to the inherent wearability needs of prosthetic applications and, in addition, they require a considerably higher computational power/cost; the nonimage-based technologies are based on inertial measurement units, which usually require specific limbs motion, thus interfering with the final limb actions to be performed or requiring higher coordination efforts even to perform simple tasks.

The use of hybrid approaches has also experienced considerable growth. Undoubtedly, the combined use of different biosignals could help compensate for the weaknesses of each single biosignal, thus leading to improved performances. On the other hand, however, this is achieved at higher costs in terms of both hardware and software complexities. Therefore, the pros and cons of hybrid approaches should be carefully evaluated and may vary among different targets. Indeed, their use in the control of robots, smart environments, and virtual realities is still limited. In contrast, in prosthetic control, gesture recognition, and communication, their use has reached or overcome the use of other biosignals. In particular, hybrid technologies have been used in 40% of all studies focused on communication HMIs, thus overcoming all other biosignals. This result suggests that the considerable complexity of communication tasks benefited from combining different biosignals much more than other target applications.

## 5. Conclusions

This survey outlines the developments and research trends in biosignal-based Human–Machine Interfaces designed for assistive and rehabilitation purposes. Recently, several patents have filed for assistance and rehabilitation devices, demonstrating the ever-growing interest in developing new technologies by companies, universities, and research institutes. Some examples are: an ambulation exoskeleton for limb rehabilitation in patients suffering from poststroke motor disabilities; a lower limb wearable robotic system able to support the flexion/extension movements of the hip and knee joints for rehabilitation applications; a hand exoskeleton for use in robot-assisted rehabilitation; a vision rehabilitation system based on the remote control of the physician; a grip assistance device based on functional electrical stimulation (FES), which perceives the movement intentions by the EMG signal from a paretic limb and provides electrical stimuli in order to activate the muscles involved in grasp actions; etc. [223].

In conclusion, biopotential-based HMIs are involved in all considered targets. Their major drawback is the need for stable electrical contact over time. Among these HMIs, BCI is a promising exploration area and could be crucial for people with severe disabilities. However, this technology is not mature enough to be reliably used in daily life environments.

In the last several years, many alternatives to biopotentials have been available in the field of assistive and rehabilitative HMIs. Recently, research on prosthetic control has been strongly directed towards using muscle mechanical motion as an EMG alternative; it has also been widely tested for all the other targets considered. HMIs that exploit this kind of biosignals achieve comparable performances to EMG, overcoming its well-known limitations. Nonetheless, they do require the user to have adequate muscle contraction capacity. Body motion-based HMIs are mainly used for smart environment and virtual reality control. The use of image-based and nonimage-based techniques turned out to be well balanced. Hybrid HMIs represent an emergent trend and have already provided a remarkable contribution to the communication target. They could help compensate for single biosignals’ weaknesses but must be adapted to specific applications.

The future will probably be characterized by the development of interactive/intelligent systems that allow any person, even someone affected by severe motor disabilities, to provide commands to a wide range of machines. Shared control, predictable machine learning techniques, closed-loop control based on sensory feedback, and human-oriented design requirements will be the critical challenges for future research. In addition, the HMIs design, especially in the field of assistive technologies, would probably be directed towards the development of “universal interfaces” [224], capable of recognizing and executing any user command (e.g., home automation devices, smart wheelchairs, personal communicators, etc.) in order to let these systems to be easily used by all kinds of users, regardless of age, languages, and degree of disability.

## Figures and Tables

**Table 1:** Review studies about HMIs for assistive and rehabilitation purposes (starting from 2015 to 2021).

Authors [Reference]	Title	Topic
Taylor et al. [18]	The use of gaming technology for rehabilitation in people with multiple sclerosis	Exergaming
De Gama et al. [29]	Motor Rehabilitation Using Kinect: A Systematic Review	Exergaming
Laver et al. [30]	Virtual reality for stroke rehabilitation	Exergaming
Wright et al. [23]	A Review of Control Strategies in Closed-Loop Neuroprosthetic Systems	Prosthetic control
Ciancio et al. [24]	Control of Prosthetic Hands via the Peripheral Nervous System	Prosthetic control
Frisoli et al. [22]	New generation emerging technologies for neurorehabilitation and motor assistance	Wearable devices (exoskeletons)
Rosly et al. [31]	Exergaming for individuals with neurological disability: A systematic review	Exergaming
Lazarou et al. [5]	EEG-Based Brain–Computer Interfaces for Communication and Rehabilitation of People with Motor Impairment: A Novel Approach of the 21st Century	BCI
Ngan et al. [25]	Strategies for neural control of prosthetic limbs: From electrode interfacing to 3D printing	Prosthetic control
Parajuli et al. [26]	Real-Time EMG Based Pattern Recognition Control for Hand Prostheses: A Review on Existing Methods, Challenges, and Future Implementation	Prosthetic control
Igual et al. [27]	Myoelectric Control for Upper Limb Prostheses	Prosthetic control
Kumar et al. [28]	Prosthetic hand control: A multidisciplinary review to identify strengths, shortcomings, and the future	Prosthetic control
Reis et al. [32]	Exergames for motor rehabilitation in older adults: An umbrella review	Exergaming
Garcia-Agundez et al. [20]	Recent advances in rehabilitation for Parkinson’s Disease with exergames: A Systematic Review	Exergaming
Fatima et al. [19]	Intracortical brain–machine interfaces for controlling upper-limb-powered muscle and robotic systems in spinal cord injury	Prosthetic control
Grushko et al. [10]	Control Methods for Transradial Prostheses Based on Remnant Muscle Activity and Its Relationship with Proprioceptive Feedback	Prosthetic control
Mohebbi et al. [21]	Human–Robot Interaction in Rehabilitation and Assistance: A Review	Robotic control
Ptito et al. [6]	Brain–Machine Interfaces to Assist the Blind	BCI
Li et al. [33]	Gesture Recognition Using Surface Electromyography and Deep Learning for Prostheses Hand: State-of-the-Art, Challenges, and Future	Prosthetic control
Ahmadizadeh et al. [9]	Human Machine Interfaces in Upper-Limb Prosthesis Control: A Survey of Techniques for Preprocessing and Processing of Biosignals	Prosthetic control
Baniqued et al. [7]	Brain–computer interface robotics for hand rehabilitation after stroke: A systematic review	BCI

**Table 2:** EEG-based HMIs.

Authors [Reference]	Kind of Biopotential	Target	Field
Gao et al. [46]	Scalp EEG	Prosthetic Control	Assistance
Gannouni et al. [47]	Scalp EEG	Prosthetic Control	Assistance, Rehabilitation
Fuentes-Gonzalez et al. [48]	Scalp EEG	Prosthetic Control	Assistance
Song et al. [49]	Scalp EEG	Robotic Control	Assistance, Rehabilitation
Korovesis et al. [50]	Scalp EEG	Robotic Control	Assistance
Antoniou et al. [64]	Scalp EEG	Robotic Control	Rehabilitation
Xu et al. [14]	Scalp EEG	Robotic Control	Assistance, Rehabilitation
Liang et al. [34]	Scalp EEG	Robotic Control	Assistance, Rehabilitation
Matsushita et al. [65]	ECoG	Robotic Control	Assistance
Spataro et al. [66]	Scalp EEG	Robotic Control	Assistance
López-Larraz et al. [67]	Scalp EEG	Robotic Control	Rehabilitation
Xu et al. [36]	Scalp EEG	Robotic Control	Rehabilitation
Kwak et al. [82]	Scalp EEG	Robotic Control	Rehabilitation
Hortal et al. [68]	Scalp EEG	Robotic Control	Assistance, Rehabilitation
Varada et al. [15]	Scalp EEG	Robotic Control, Smart Environment Control	Assistance, Rehabilitation
Wang et al. [69]	Scalp EEG	Robotic Control, Prosthetic Control	Rehabilitation
Zhan Hong et al. [70]	Scalp EEG	Prosthetic Control	Assistance
Ortiz et al. [71]	Scalp EEG	Robotic Control	Assistance, Rehabilitation
Kasim et al. [72]	Scalp EEG	Prosthetic Control	Assistance
Murphy et al. [73]	Scalp EEG	Prosthetic Control	Assistance
Li et al. [74]	sEEG	Prosthetic Control	Assistance
Bhagat et al. [75]	Scalp EEG	Robotic Control	Rehabilitation
Morishita et al. [76]	ECoG	Prosthetic Control	Rehabilitation
Zhang et al. [77]	Scalp EEG	Prosthetic Control	Assistance, Rehabilitation
Yanagisawa et al. [78]	ECoG	Prosthetic Control	Assistance, Rehabilitation
He et al. [35]	Scalp EEG	Robotic Control	Rehabilitation
Tang et al. [79]	Scalp EEG	Robotic Control	Assistance
Randazzo et al. [80]	Scalp EEG	Robotic Control	Assistance, Rehabilitation
Li et al. [81]	Scalp EEG	Robotic Control	Assistance, Rehabilitation
Araujo et al. [83]	Scalp EEG	Robotic Control	Rehabilitation
Kashihara et al. [84]	Scalp EEG	Communication	Assistance
Mahmoudi and Erfanian [85]	Scalp EEG	Prosthetic Control	Assistance

**Table 3:** EMG-based HMIs.

Authors [Reference]	Kind of Biopotential	Target	Field
Eisenberg et al. [53]	sEMG	Gesture Recognition, Prosthetic Control	Assistance
Tavakoli et al. [54]	sEMG	Gesture Recognition, Prosthetic Control	Assistance
Bai et al. [87]	sEMG	Gesture Recognition, Prosthetic Control	Assistance
Cao et al. [88]	sEMG	Prosthetic Control	Assistance
Benatti et al. [89]	sEMG	Gesture Recognition, Prosthetic Control	Assistance
Ulloa et al. [90]	sEMG	Prosthetic Control	Assistance
Polisiero et al. [91]	sEMG	Prosthetic Control	Assistance
Gailey et al. [92]	sEMG	Gesture Recognition, Prosthetic Control	Assistance
Bernardino et al. [93]	sEMG	Gesture Recognition, Prosthetic Control	Assistance
Zhao et al. [94]	sEMG	Gesture Recognition, Prosthetic Control	Assistance
Carrozza et al. [95]	sEMG	Prosthetic Control	Assistance
Jiang et al. [96]	sEMG	Gesture Recognition, Prosthetic Control	Assistance
Brunelli et al. [97]	sEMG	Gesture Recognition, Prosthetic Control	Assistance
Shair et al. [98]	sEMG	Prosthetic Control	Assistance
Khushaba et al. [99]	sEMG	Gesture Recognition, Prosthetic Control	Assistance
Kamavuako et al. [100]	imEMG	Prosthetic Control	Assistance
Dewald et al. [101]	imEMG	Gesture Recognition, Prosthetic Control, Virtual Reality Control	Assistance
Al-Timemy et al. [102]	sEMG	Gesture Recognition, Prosthetic Control	Assistance
Zhang et al. [103]	sEMG	Prosthetic Control	Assistance
Dalley et al. [104]	sEMG	Prosthetic Control	Assistance
Russo et al. [105]	sEMG	Gesture Recognition, Prosthetic Control	Assistance
Stepp et al. [106]	sEMG	Prosthetic Control	Rehabilitation
Visconti et al. [107]	sEMG	Gesture Recognition, Prosthetic Control, Robotic Control, Smart Environment Control, Virtual Reality Control	Assistance, Rehabilitation
Lu and Zhou [108]	sEMG	Smart Environment Control	Assistance
Kumar et al. [109]	sEMG	Robotic Control	Assistance
Kalani et al. [110]	sEMG	Robotic Control	Rehabilitation
Alibhai et al. [39]	sEMG	Gesture Recognition, Robotic Control	Assistance
Fall et al. [37]	sEMG	Robotic Control	Assistance
Song et al. [40]	sEMG	Gesture Recognition, Robotic Control	Assistance
Laksono et al. [38]	sEMG	Robotic Control	Assistance
Xu et al. [41]	sEMG	Robotic Control	Assistance
Zhang et al. [111]	sEMG	Robotic Control	Assistance
Hamedi et al. [112]	sEMG	Gesture Recognition	Assistance, Rehabilitation
Wege and Zimmermann [113]	sEMG	Robotic Control	Rehabilitation
Ho et al. [114]	sEMG	Robotic Control	Rehabilitation
Loconsole et al. [115]	sEMG	Robotic Control	Rehabilitation
Hussain et al. [116]	sEMG	Gesture Recognition, Robotic Control	Assistance
Abdallah et al. [117]	sEMG	Robotic Control	Rehabilitation
Secciani et al. [118]	sEMG	Robotic Control	Assistance
Song et al. [119]	sEMG	Robotic Control	Rehabilitation
Liu et al. [120]	sEMG	Robotic Control	Rehabilitation
Cai et al. [121]	sEMG	Robotic Control	Rehabilitation
Yin et al. [122]	sEMG	Robotic Control	Rehabilitation
Tang et al. [123]	sEMG	Robotic Control	Rehabilitation
Lu et al. [124]	sEMG	Robotic Control	Rehabilitation
Gui et al. [125]	sEMG	Robotic Control	Rehabilitation
La Scaleia et al. [126]	sEMG	Robotic Control, Virtual Reality Control	Assistance, Rehabilitation
Lyu et al. [127]	sEMG	Robotic Control	Rehabilitation

**Table 4:** ENG-based HMIs.

Authors [Reference]	Target	Field
Noce et al. [52]	Gesture Recognition, Prosthetic Control	Assistance
Nguyen et al. [55]	Prosthetic Control	Assistance
Noce et al. [59]	Gesture Recognition, Prosthetic Control	Assistance

**Table 5:** EOG-based HMIs.

Authors [Reference]	Target	Field
Golparvar and Yapici [56]	Robotic Control, Smart Environment Control	Assistance
Zhang et al. [42]	Smart Environment Control	Assistance
Huang et al. [57]	Robotic Control	Assistance
Martínez-Cerveró et al. [128]	Communication	Assistance
Perez Reynoso et al. [129]	Robotic Control	Assistance
Choudhari et al. [130]	Robotic Control	Assistance
Heo et al. [131]	Communication, Robotic Control	Assistance
Guo et al. [132]	Smart Environment Control	Assistance
Wu et al. [133]	Robotic Control, Smart Environment Control	Assistance, Rehabilitation

**Table 6:** Hybrid biopotential-based HMIs.

Authors [Reference]	Kind of Biopotential	Target	Field
Gordleeva et al. [51]	EEG + EMG	Robotic Control	Rehabilitation
Ferreira et al. [134]	EEG + EMG	Robotic Control	Assistance
Zhang et al. [135]	EEG + EMG + EOG	Gesture Recognition, Robotic Control	Assistance, Rehabilitation
Huang et al. [136]	EEG + EOG	Robotic Control	Assistance
Ma et al. [12]	EEG + EOG	Robotic Control	Assistance
Ma et al. [137]	EEG + EOG	Robotic Control	Assistance
Arrow et al. [58]	EMG + ERG	Prosthetic Control	Assistance
Rezazadeh et al. [138]	EEG + EMG	Virtual Reality Control	Assistance
Rezazadeh et al. [139]	EEG + EMG + EOG	Communication, Gesture Recognition	Assistance, Rehabilitation
Iáñez et al. [140]	EEG + EOG	Smart Environment Control	Assistance
Laport et al. [141]	EEG + EOG	Smart Environment Control	Assistance
Neto et al. [142]	EEG + EMG + EOG	Robotic Control	Assistance

**Table 7:** Muscle gross motion-based HMIs.

Authors [Reference]	Kind of sensor	Application Site	Target	Field
Prakash et al. [144]	FSR	Forearm	Prosthetic Control	Assistance
Clemente et al. [165]	Magnetic Field	Forearm	Prosthetic Control	Assistance
Xiao et al. [152]	FSR	Forearm	Robotic Control	Rehabilitation
Ferigo et al. [153]	FSR	Forearm	Prosthetic Control	Assistance
Esposito et al. [154]	FSR	Forearm	Gesture Recognition, Prosthetic Control	Assistance
Esposito et al. [155]	FSR	Forearm	Prosthetic Control	Assistance
Esposito et al. [156]	FSR	Forearm	Prosthetic Control	Assistance
Ha et al. [157]	Piezoelectric	Forearm	Prosthetic Control	Assistance
Ha et al. [158]	Piezoelectric	Forearm	Prosthetic Control	Assistance
Ahmadizadeh et al. [151]	FSR	Forearm	Prosthetic Control	Assistance
Fujiwara et al. [159]	Optical Fibre	Forearm	Gesture Recognition, Prosthetic Control, Virtual Reality Control	Assistance, Rehabilitation
Bifulco et al. [160]	Resistive	Forearm	Prosthetic Control	Assistance
Radmand et al. [161]	FSR	Forearm	Prosthetic Control	Assistance
Cho et al. [162]	FSR	Forearm	Prosthetic Control	Assistance
Dong et al. [163]	Triboelectric	Hand	Robotic Control, Virtual Reality Control	Assistance, Rehabilitation
Zhu et al. [16]	Triboelectric	Hand	Robotic Control, Virtual Reality Control	Assistance, Rehabilitation
An et al. [164]	Triboelectric	Arm	Robotic Control, Smart Environment Control	Assistance
Tarantino et al. [166]	Magnetic field	Forearm	Prosthetic Control	Assistance
Kumar et al. [167]	Piezoresistive	Hand	Communication, Smart Environment Control	Assistance
Castellini et al. [168]	Resistive	Forearm	Prosthetic Control	Assistance
Dong et al. [169]	Piezoelectric	Wrist	Prosthetic Control	Assistance
Lim et al. [170]	Piezoelectric	Forearm, Wrist	Robotic Control	Assistance
Rasouli et al. [171]	Piezoelectric	Forearm	Prosthetic Control	Assistance

**Table 8:** Muscle vibrations-based HMIs.

Authors [Reference]	Kind of Sensor	Application Site	Target	Field
Asheghabadi et al. [146]	Piezoelectric + Strain Gauge	Forearm	Prosthetic Control	Assistance
Castillo et al. [176]	Microphone	Forearm	Prosthetic Control	Assistance
Wicaksono et al. [177]	Piezoresistive	Lower limb	Prosthetic Control, Robotic Control	Assistance, Rehabilitation
Xie et al. [178]	Accelerometer	Forearm	Gesture Recognition, Prosthetic Control	Assistance

**Table 9:** Muscle–tendons movement-based HMIs.

Authors [Reference]	Kind of Sensor	Application Site	Target	Field
Wu et al. [145]	Bioamplifier	Forearm	Gesture Recognition, Prosthetic Control	Assistance
Chen et al. [147]	US probe	Forearm	Prosthetic Control	Assistance
Huang et al. [179]	US probe	Forearm	Gesture Recognition, Prosthetic Control, Robotic Control	Assistance
Li et al. [180]	US transducer	Forearm	Gesture Recognition, Robotic Control	Rehabilitation
Ortenzi et al. [181]	US probe	Forearm	Prosthetic Control	Assistance
Sikdar et al. [182]	US probe	Forearm	Prosthetic Control	Assistance
Sierra González et al. [183]	US probe	Forearm	Robotic Control	Rehabilitation
Castellini et al. [184]	US probe	Forearm	Robotic Control	Rehabilitation
Shi et al. [185]	US probe	Forearm	Prosthetic Control	Assistance

**Table 10:** Hybrid muscle mechanical motion-based HMIs.

Authors [Reference]	Kind of Sensor	Application Site	Target	Field
Esposito et al. [143]	FSR	Forearm	Prosthetic Control	Assistance
Booth et al. [186]	Piezoelectric	Wrist	Gesture Recognition, Prosthetic Control, Robotic Control, Smart Environment Control, Virtual Reality Control	Assistance, Rehabilitation

**Table 11:** Image-based body motion HMIs.

Authors [Reference]	Tracked Body Part	Target	Field
Maule et al. [187]	Eyes	Robotic Control	Assistance
Bissoli et al. [43]	Eyes	Smart Environment Control	Assistance
Lin et al. [188]	Eyes	Smart Environment Control	Assistance
Conci et al. [189]	Hands	Gesture Recognition, Smart Environment Control	Assistance
Baklouti et al. [190]	Head/Mouth	Robotic control	Rehabilitation
Chang et al. [191]	Head	Communication	Assistance
Gautam et al. [192]	Head	Robotic Control	Assistance
Gmez-Portes et al. [193]	Whole body	Virtual Reality Control	Rehabilitation
Palaniappan et al. [194]	Upper limb	Virtual Reality Control	Rehabilitation
Nguyen et al. [195]	Whole body with “JRS”; wrist and elbow with “MHT”	Virtual Reality Control	Rehabilitation

**Table 12:** Nonimage-based body motion HMIs.

Authors [Reference]	Kind of Sensors	Application Sites of Sensors	Target	Field
Chuang et al. [196]	Resistive flex sensors	Embedded in a glove	Gesture Recognition	Assistance
Dong et al. [197]	Piezoresistive strain sensors (based on PDMS-CB)	Embedded in a glove	Gesture Recognition, Robotic Control	Assistance, Rehabilitation
Zhu et al. [198]	Stretchable conductive yarns	Embedded in a glove	Robotic Control, Smart Environment Control	Assistance
Hang et al. [203]	PAAm hydrogel-based strain sensor	Various body positions	Gesture Recognition, Robotic Control	Assistance
Ueki et al. [199]	Force/torque sensors and 3D motion sensor	Embedded in a glove, hand and forearm	Robotic Control, Virtual Reality Control	Rehabilitation
Rahman et al. [200]	Flex sensors	Embedded in a glove, hand	Robotic Control	Rehabilitation
Cortese et al. [201]	MEMS accelerometers	Embedded in a glove, hand	Robotic Control	Rehabilitation
Han et al. [202]	Three-axis gyroscope	Hand back	Gesture Recognition, Smart Environment Control	Assistance

**Table 13:** Hybrid controls for HMIs based on Biopotentials and Image-based systems.

Authors [Reference]	Kind of Sensors	Application Site of Electrodes	Location of Video System/s	Target	Field
Wei and Hu [204]	EMG electrodes + Video camera	Forehead	towards the subject’s face	Robotic Control	Assistance
Haung et al. [205]	Video camera + EEG electrodes	10–20 EEG international system	towards the subject’s face	Communication	Assistance
Downey et al. [206]	Intracortical microelectrode arrays + RGB–D camera	Motor cortex	on the arm of the robot	Robotic Control	Assistance
Bu et al. [207]	EMG electrodes + Video camera	Forearm	towards the target objects	Prosthetic Control	Assistance
Malechka et al. [208]	EEG electrodes + 3 video cameras	10–10 EEG international system	two video cameras towards subject’s face (one for each eye tracking); one video camera towards the target objects	Smart Environment Control	Assistance
McMullen et al. [209]	ECoG and depth electrodes + Microsoft Kinect + video camera	Motor cortex	Kinect sensor towards the target objects; video camera towards the subject’s face	Prosthetic Control	Assistance
Frisoli et al. [210]	EEG electrodes + scene camera (i.e., 2 infrared cameras + 2 infrared LEDs + 1 wide-angle camera) + Microsoft Kinect	Over sensorimotor cortex	Scene camera mounted on glasses; Kinect sensor towards the target objects	Robotic Control	Rehabilitation

**Table 14:** Hybrid controls for HMIs based on Biopotentials and Mechanical Motion Detection.

Authors [Reference]	Kind of Hybrid Sensors	Application Sites of Hybrid Sensors	Target	Field
Dunai et al. [217]	sEMG electrodes + FSR sensors	sEMG electrodes on Extensor digitorum (forearm). FSR sensors on prosthetic fingertips.	Prosthetic Control	Assistance
Krasoulis et al. [211]	Hybrid sEMG/IMU sensors	Eight hybrid sensors are equally spaced around the forearm (3 cm below the elbow); two are placed on the extrinsic hand muscles superficialis; two are placed on the biceps and triceps brachii muscles.	Prosthetic Control	Assistance
Shahzad et al. [212]	sEMG electrodes + IMU	Two sEMG sensors are placed on the forearm flexors, and other two are placed at the forearm extensors. The forearm IMU was placed proximal to the wrist, and the upper arm IMU was paced over the biceps brachii muscle.	Gesture Recognition, Prosthetic control	Assistance
Kyranou et al. [213]	Hybrid sEMG/IMU	Twelve hybrid sensors are placed on the proximal forearm via an elastic bandage.	Gesture Recognition, Prosthetic control	Assistance
Jaquier et al. [214]	sEMG electrodes + pressure sensors (resistive elastomers)	Ten sEMG sensors are placed on the proximal forearm. Ten pressure sensors (via a bracelet) are placed on the proximal forearm.	Gesture Recognition, Prosthetic control	Assistance
Guo et al. [215]	Hybrid sEMG/NIRS sensors	Four hybrid sensors are attached above flexor carpi ulnaris, flexor carpi radialis, extensor carpi radialis longus, and extensor digitorum.	Gesture Recognition, Virtual Reality Control	Assistance
Xia et al. [13]	Hybrid sEMG/US sensors	Four hybrid sensors are mounted on the forearm by means of an armband.	Gesture Recognition, Prosthetic Control	Assistance
Dwivedi et al. [216]	sEMG + FSR sensors	Three EMG sensors are embedded in a sleeve. Five FSR sensors are embedded in a sleeve.	Robotic Control	Assistance

**Table 15:** Hybrid controls for HMIs based on various combinations of sensors.

Authors [Reference]	Kind of Hybrid Sensors	Application Sites of Hybrid Sensors	Target	Field
Ubeda et al. [11]	EEG electrodes + RFID tags	10–20 EEG international system; RFID tags near by the target objects.	Robotic Control	Assistance, Rehabilitation
Perez et al. [218]	Video camera + IMU sensor	Video camera (webcam) towards the patient’s face; IMU sensor mounted on a cap or headband.	Robotic Control	Assistance
Bastos-Filho et al. [219]	EMG electrodes + video cameras + IMU sensor + pressure sensor + EEG electrodes	EMG electrodes on temporal muscles; Video camera on a pair of glasses worn by the user; IMU sensor mounted on a cap; Pressure sensor into a straw; 10–20 EEG international system; Video camera towards the user’s face.	Robotic Control	Assistance
Anwer et al. [220]	Microphone + video camera	Microphone embedded in the wheelchair; Video camera towards the user’s face.	Robotic Control	Assistance
Gardner et al. [221]	Acoustic MMG sensor + IMU sensor + video camera	MMG and IMU (embedded in a compression sleeve) on the biceps; Video camera on a pair of glasses worn by the user.	Prosthetic Control	Assistance
Wu et al. [222]	EOG electrodes + switches (push button, InfraRed, mercury, long–short tone, and pacifier)	EOG electrodes on eyebrow arch; Various switches are positioned to be activated by the user.	Communication	Assistance

## Data Availability

The data presented in this study are available on request from the corresponding author.
